# KDM6 Demethylase Independent Loss of Histone H3 Lysine 27 Trimethylation during Early Embryonic Development

**DOI:** 10.1371/journal.pgen.1004507

**Published:** 2014-08-07

**Authors:** Karl B. Shpargel, Joshua Starmer, Della Yee, Michael Pohlers, Terry Magnuson

**Affiliations:** Department of Genetics, Carolina Center for Genome Sciences, and Lineberger Comprehensive Cancer Center, University of North Carolina, Chapel Hill, North Carolina, United States of America; Massachusetts General Hospital, Howard Hughes Medical Institute, United States of America

## Abstract

The early mammalian embryo utilizes histone H3 lysine 27 trimethylation (H3K27me3) to maintain essential developmental genes in a repressive chromatin state. As differentiation progresses, H3K27me3 is removed in a distinct fashion to activate lineage specific patterns of developmental gene expression. These rapid changes in early embryonic chromatin environment are thought to be dependent on H3K27 demethylases. We have taken a mouse genetics approach to remove activity of both H3K27 demethylases of the *Kdm6* gene family, *Utx* (*Kdm6a*, X-linked gene) and *Jmjd3* (*Kdm6b*, autosomal gene). Male embryos null for active H3K27 demethylation by the *Kdm6* gene family survive to term. At mid-gestation, embryos demonstrate proper patterning and activation of *Hox* genes. These male embryos retain the Y-chromosome UTX homolog, UTY, which cannot demethylate H3K27me3 due to mutations in catalytic site of the Jumonji-C domain. Embryonic stem (ES) cells lacking all enzymatic KDM6 demethylation exhibit a typical decrease in global H3K27me3 levels with differentiation. Retinoic acid differentiations of these ES cells demonstrate loss of H3K27me3 and gain of H3K4me3 to *Hox* promoters and other transcription factors, and induce expression similar to control cells. A small subset of genes exhibit decreased expression associated with reduction of promoter H3K4me3 and some low-level accumulation of H3K27me3. Finally, *Utx* and *Jmjd3* mutant mouse embryonic fibroblasts (MEFs) demonstrate dramatic loss of H3K27me3 from promoters of several *Hox* genes and transcription factors. Our results indicate that early embryonic H3K27me3 repression can be alleviated in the absence of active demethylation by the *Kdm6* gene family.

## Author Summary

H3K27me3 represses developmental genes at initial embryonic stages. The KDM6 family, comprised of UTX and JMJD3, are the only known proteins that demethylate H3K27me3 and they are hypothesized to catalyze the rapid removal of repressive chromatin in early mammalian development. However, we report that male embryos carrying mutations in both *Utx* and *Jmjd3* survive to term and appear phenotypically normal at mid-gestation. We utilize several cell culture models to demonstrate that H3K27me3 is lost from repressed promoters in the absence of active KDM6 demethylation. Our data indicate that KDM6 H3K27me3 demethylation is not essential in the early embryo and that H3K27me3 loss from developmental genes occurs via novel mechanisms.

## Introduction

The mammalian embryo undergoes drastic changes in cellular specification and gene expression programs throughout development. These changes are facilitated by post-translational modifications to histones, which provide an epigenetic mechanism to coordinate initiation and maintenance of lineage specific transcriptional profiles that can be inherited through multiple cellular divisions. In embryonic stem cells and other pluripotent progenitors, crucial developmental genes are maintained in a quiescent state. A bivalent epigenetic signature defines this large class of genes. These promoters are maintained in a repressive chromatin state through histone H3 lysine 27 trimethylation (H3K27me3), however the presence of an active chromatin modification (H3K4me3) suggests that these genes are poised for rapid induction as development dictates [1]–[4]. Bivalent promoters have been identified in ES cells, the early embryo, lineage progenitors, and the germline [4]–[15]. With specification or differentiation, these bivalent promoters can be resolved to either a univalent H3K4me3 active state or a H3K27me3 repressed state. In numerous cell culture model systems, histone demethylases are required to remove H3K27me3 to promote gene activation, suggesting that H3K27me3 demethylation is essential in embryonic development [16]–[30].

H3K27me3 demethylases are members of the KDM6, Jumonji-C (JmjC) domain family of histone demethylases. The three KDM6 proteins, JMJD3 (KDM6B, encoded by an autosomal gene), UTX (KDM6A, X-chromosome), and UTY (Y-chromosome), all share a well-conserved JmjC histone demethylation domain [31]. Within this protein family, JMJD3 and UTX demethylate H3K27 tri-methyl and di-methyl residues, whereas human UTY demonstrates greatly reduced catalytic activity [27], [31]–[34]. Mouse UTY, despite maintaining 82% similarity to the X-chromosome homologue UTX, does not demethylate H3K27me3 due to mutations in the catalytic active site of its JmjC domain [31].

UTX and JMJD3 are individually involved in early embryonic specification events in cell culture [17]–[19], [22], [32], leading to the hypothesis that H3K27me3 demethylases function in early embryonic differentiation events. However, mouse mutagenesis suggests otherwise, as embryos deficient for individual demethylases survive to term. *Jmjd3^−/−^* homozygotes exhibit post-natal lethality due to neonatal respiratory deficits [35]. *Utx^−/y^* hemizygous males survive to adulthood and exhibit a normal lifespan [31]. In contrast, mutation of the Polycomb Repressive Complex 2 (PRC2) that methylates H3K27 yields precocious expression of early embryonic developmental genes and arrest in gastrulation [36]–[39]. *Utx^−/−^* homozygous females and *Utx^−^;Uty^−^* hemizygous males are both mid-gestational lethal with developmental delay and defects in embryonic heart development [31]. Therefore, the mid-gestational cardiovascular lethality that is driven by loss of UTX/UTY is due to demethylase independent function of these proteins. It is not clear if an early embryonic demethylase dependent function exists for the KDM6 family as some redundancy may exist between JMJD3 and UTX.

To study the role of the KDM6 family in early embryonic development we generated mutations designed to eliminate all KDM6 H3K27me3 demethylase activity in the developing mouse embryo. Male *Utx^−/y^;Jmjd3^−/−^* embryos devoid of KDM6 H3K27 demethylation survived to term. Mid-gestational *Utx^−/y^;Jmjd3^−/−^* embryos appeared phenotypically normal with characteristic features of embryonic day 10.5 (E10.5) embryos. We utilized several model systems (embryoid body, retinoic acid, mouse embryonic fibroblasts) to demonstrate that H3K27me3 can be removed from the promoters of repressed genes in the absence of active KDM6 demethylation. We conclude that KDM6 demethylases are not essential for early embryonic development and that H3K27me3 repression can be alleviated in the absence of active KDM6 demethylation.

## Results

### Mouse embryos devoid of KDM6 demethylation survive to term and display normal early embryonic phenotypes

**Figure 1:**
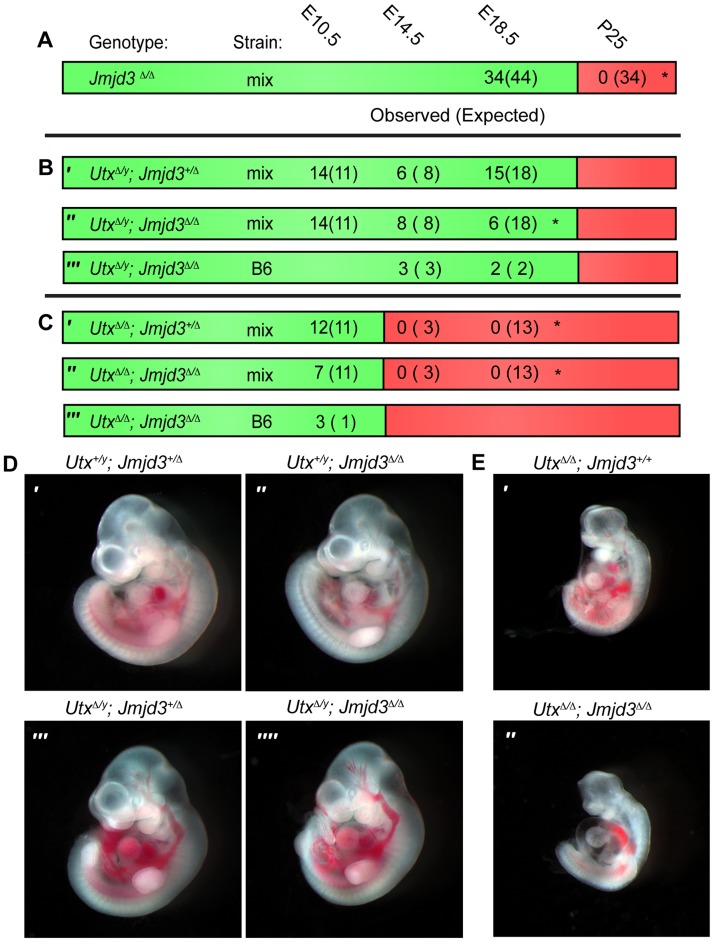
*Utx* and *Jmjd3* mutant phenotypes. (A–C) Observed and Expected (in parentheses) numbers of indicated genotypes at embryonic (day 10.5, 14.5 or 18.5) or postnatal (day 25, weaning) timepoints. Data are included for *Jmjd3* ^Δ/Δ^ (A), *Utx* ^Δ/*y*^;*Jmjd3* ^*+/*Δ^ (B′), *Utx* ^Δ/*y*^;*Jmjd3* ^Δ/Δ^ (B″ and B′″), *Utx* ^Δ/Δ^;*Jmjd3* ^+/^^Δ^ (C′), and *Utx* ^Δ/Δ^;*Jmjd3* ^Δ/Δ^ (C″ and C′″) genotypes. Green regions denote viability and red denotes lethality. Mix denotes a mixed genetic background and B6 has been backcrossed to C57BL6 for >5 generations. Significant deviations from expected allele frequencies as determined by χ^2^ p-value are: (A) *<0.001, (B) * = 0.005, and (C) *<0.001. (D,E) Embryonic day 10.5 images of (D) male *Utx^+/y^*;*Jmjd3* ^+/Δ^ (D′), *Utx^+/y^*;*Jmjd3* ^Δ/Δ^ (D″), *Utx* ^Δ/^ ^*y*^;*Jmjd3* ^*+/*Δ^ (D′″), *Utx* ^Δ/^ ^*y*^;*Jmjd3* ^Δ/Δ^ (D″″) embryos and (E) female *Utx* ^Δ/Δ^ (E′) and *Utx* ^Δ/Δ^;*Jmjd3* ^Δ/Δ^ (E″) embryos, B6 background.

To remove H3K27 demethylase activity in the mouse embryo we generated mutant alleles in both *Utx* and *Jmjd3*. We previously characterized the generation of the *Utx^fl^* allele that flanks exon 3 with loxP sites [31]. Cre mediated deletion of exon 3 (*Utx* ^Δ^) created a frameshift in the coding sequence and is null for UTX protein. We now characterize a targeted allele, *Jmjd3^tm1Mag^* (*Jmjd3^fl^*) that integrates loxP sites 5′ to exon 14 and 3′ to exon 20 (Figure S1A). As verified by Southern blotting, PCR genotyping, and RT-PCR (Figure S1B, S1C, and S1D), Cre mediated deletion of this portion of the coding sequence (*Jmjd3* ^Δ^) removed the JmjC catalytic H3K27 demethylase domain (Figure S1A). Similar to published reports, *Jmjd3* ^Δ/Δ^ homozygous pups died at birth with respiratory defects (Figure S1E). *Jmjd3* ^Δ/Δ^ homozygous embryos appeared phenotypically normal at mid-gestation (Figure S1F); however, several phenotypes manifested late in embryonic development which will be described elsewhere. While *Jmjd3* ^Δ/Δ^ homozygous pups were not observed at weaning, they were readily recovered at E18.5 (Figure 1A).

We next attempted to derive *Utx^Δ/y^*;*Jmjd3^Δ/Δ^* embryos whereby all KDM6 H3K27 demethylation is lost, while retaining the demethylase independent function of wild-type UTY. Similar to *Utx^−/y^* mutation alone [31], both *Utx^Δ/y^*;*Jmjd3^+/Δ^* and *Utx^Δ/y^*;*Jmjd3^Δ/Δ^* embryos survived to E18.5 (Figure 1B′ and 1B″). However, there was some reduction in observed *Utx^Δ/y^*;*Jmjd3^Δ/Δ^* embryos relative to expected Mendelian frequencies. Expected genotype frequencies of *Utx^Δ/y^*;*Jmjd3^Δ/Δ^* embryos were obtained at E14.5, so some redundancy may exist between *Utx* and *Jmjd3* in late embryonic viability. At mid-gestation, all combinations of male *Utx* and *Jmjd3* mutation were largely indistinguishable from controls (Figure 1D). *Utx^Δ/y^*;*Jmjd3^Δ/Δ^* embryos demonstrated normal features of E10.5 embryos, such as normal size and somite numbers (35-40), prominent fore and hind-limb buds, and developed branchial arches (including separation of arch 1 into maxilar and mandibular components, Figure 1D″″). As *Utx^−/y^* post-natal lethality is more pronounced on the C57BL6/J (B6) background [31], we backcrossed *Utx^Δ^* and *Jmjd3^Δ^* alleles. On a B6 background, *Utx^Δ/y^*;*Jmjd3^Δ/Δ^* embryos remained viable at both E14.5 and E18.5 timepoints (Figure 1B′″). Given that deposition of maternal UTX into the Drosophila embryo contributes to demethylation activity in early development [40], we tested if deletion of the UTX and JMJD3 maternal pool enhances mouse phenotypes. *Utx^fl/Δ^*;*Jmjd3^fl/Δ^*;Vasa*Cre* female mice (with oocytes carrying deletion of *Utx* and *Jmjd3*) were crossed with *Utx^fl/y^*;*Jmjd3^fl/Δ^*;Vasa*Cre* male mice (with sperm carrying deletion of *Utx* and *Jmjd3*) and resulting E10 *Utx^Δ/y^*;*Jmjd3^Δ/Δ^* embryos (Figure S2A′) had completely recombined *Utx* and *Jmjd3* floxed alleles (Figure S2B) and phenocopied those derived from *Utx^+/Δ^*;*Jmjd3^+/Δ^* heterozygous mothers (Figure 1D″″).

*Utx* and *Jmjd3* knockout was confirmed by quantitative RT-PCR (Figure S3A), and at E10.5, *Utx^Δ/y^*;*Jmjd3^Δ/Δ^* embryos did not exhibit altered *Hox* expression levels or elevated global levels of H3K27me3 (Figure S3B,C,D). Comparative H3K27me3 immunofluorescence of E10.5 *Utx^+/y^*;*Jmjd3^+/Δ^* and *Utx^Δ/y^*;*Jmjd3^Δ/Δ^* embryos sectioned onto the same slide revealed similar H3K27me3 levels within heart myocardium (Figure S3E) and ISL1 positive motor neurons within the proximal spinal chord (Figure S3F). However, mouse embryonic fibroblasts (MEFs) derived from these embryos had minor, yet statistically significant elevations in H3K27me3 levels (Figure S3G,H). Overall, in the absence of KDM6 H3K27 demethylation, embryos can clearly survive through gastrulation and exhibit normal patterning at E10.5. Notably, the phenotypes of *Utx^Δ/Δ^*;*Jmjd3^+/Δ^* and *Utx^Δ/Δ^*;*Jmjd3^Δ/Δ^* female embryos were similar to *Utx^Δ/Δ^* homozygous mutation alone, as these embryos are all lethal after E10.5 (Figure 1C) and exhibit similar features of developmental delay (Figure 1E). Additionally, maternal loss of UTX and JMJD3 demethylation had no contribution to phenotypic severity (Figure S2A″). Taken together, our data indicate that *Utx/Uty* are epistatic to *Jmjd3*, which primarily functions in later developmental stages.

### ES cells with no KDM6 H3K27me3 demethylation have female specific differentiation defects

We established ES cell differentiation models to study the time-course of H3K27me3 demethylation in the absence of UTX and JMJD3. We utilized a *CAGGCre-ER* transgenic system that will induce allelic recombination with the addition of tamoxifen [41]. Following 2 days of tamoxifen treatment (+TX), *Utx^fl/y^*;*Jmjd3^fl/fl^*;*Cre^ER^* male or *Utx^fl/fl^*;*Jmjd3^fl/fl^*;*Cre^ER^* female ES lines demonstrated complete deletion of floxed exons and loss of endogenous protein (Figure S4A and S4B). A 140-KD background band is present in Figure S4B and is not lost in *Utx^fl/y^*;*Jmjd3^fl/fl^*;*Cre^ER^* ES +TX. To ensure that this is not an alternative *Utx* product, we analyzed its presence in *Utx^GT1/y^* ES cells [31], where any alternative products should be gene trapped. Even though *Utx^GT1/y^* ES cells did trap *Utx* transcripts preventing expression across the JmjC domain (Figure S4C) it did not affect the level of background bands (Figure S4B), indicating that these are indeed non-specific bands. Furthermore, western blot with a second, independent UTX antibody produced a clean blot with no UTX band in *Utx^fl/y^*;*Jmjd3^fl/fl^*;*Cre^ER^* ES +TX samples (Figure S4D).

**Figure 2:**
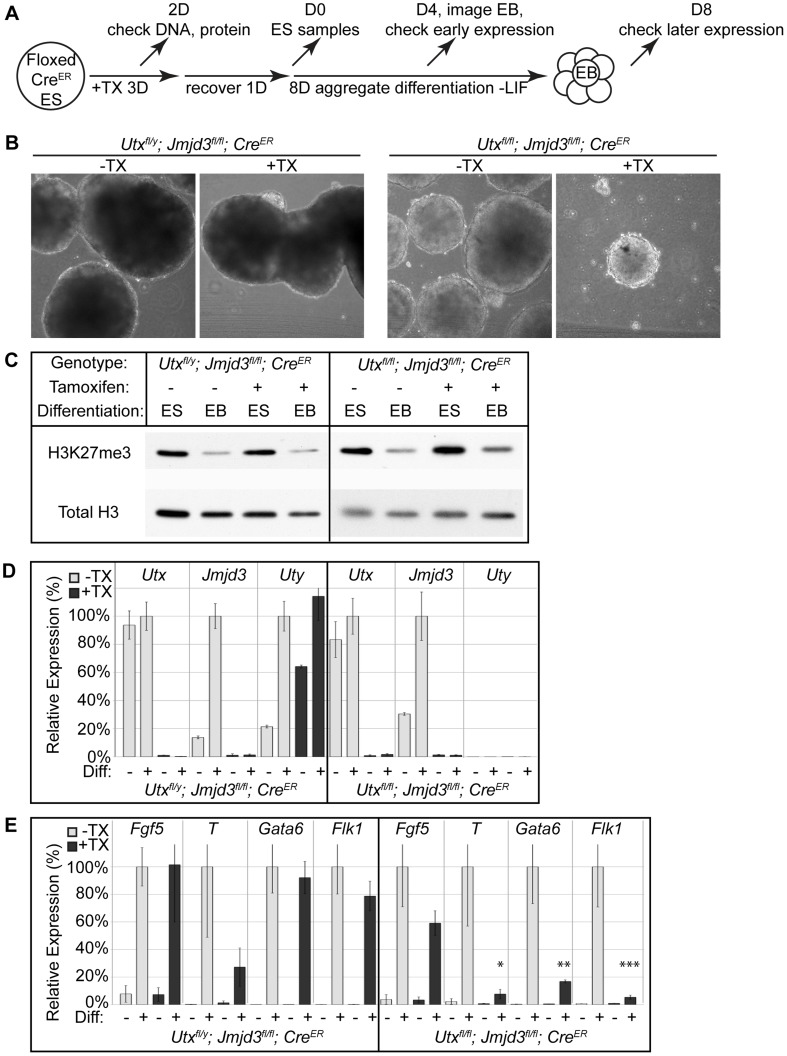
EB differentiation of male and female *Utx* and *Jmjd3* mutant ES cell lines. (A) *Utx^fl/y^*;*Jmjd3^fl/fl^*;*Cre^ER^* or *Utx^fl/fl^*;*Jmjd3^fl/fl^*;*Cre^ER^* ES cell lines were left untreated (−TX) or treated with tamoxifen for 3 days (+TX), then differentiated in aggregate suspension culture (EB). (B) Images of day 4 EBs. (C) Histones were extracted from *Utx^fl/y^*;*Jmjd3^fl/fl^*;*Cre^ER^* or *Utx^fl/fl^*;*Jmjd3^fl/fl^*;*Cre^ER^* ES cells (Differentiation ES) or day 4 EBs (Differentiation EB) left untreated (Tamoxifen −) or pre-treated with tamoxifen (Tamoxifen +) and western blotted for H3K27me3 relative to total H3 loading control. (D) Quantitative RT-PCR of *Utx*, *Jmjd3*, or *Uty* from day 0 *Utx^fl/y^*;*Jmjd3^fl/fl^*;*Cre^ER^* or *Utx^fl/fl^*;*Jmjd3^fl/fl^*;*Cre^ER^* ES cells (Differentiation −) or day 8 EBs (Differentiation +) left untreated (−TX, light grey) or pre-treated with tamoxifen (+TX, black). RT-PCR is across deleted *Utx* and *Jmjd3* exons. N = 3 samples per treatment. All samples are normalized relative to −TX Differentiation + within individual male or female lines. (E) Quantitative RT-PCR of *Fgf5* (EB day 4), *Brachyury T* (*T*, EB day 4), *Gata6* (EB day 8), or *Flk1* (EB day 8) from *Utx^fl/y^*;*Jmjd3^fl/fl^*;*Cre^ER^* or *Utx^fl/fl^*;*Jmjd3^fl/fl^*;*Cre^ER^* ES cells (Diff −) or indicated EB timepoint (Diff +) left untreated (−TX, light grey) or pre-treated with tamoxifen (+TX, black). All samples are normalized relative to −TX Differentiation + within individual male or female lines. Significant reductions in expression are indicated (T-test p-values * = 0.02, ** = 0.005, *** = 0.006, N = 3 samples per treatment).

**Figure 3:**
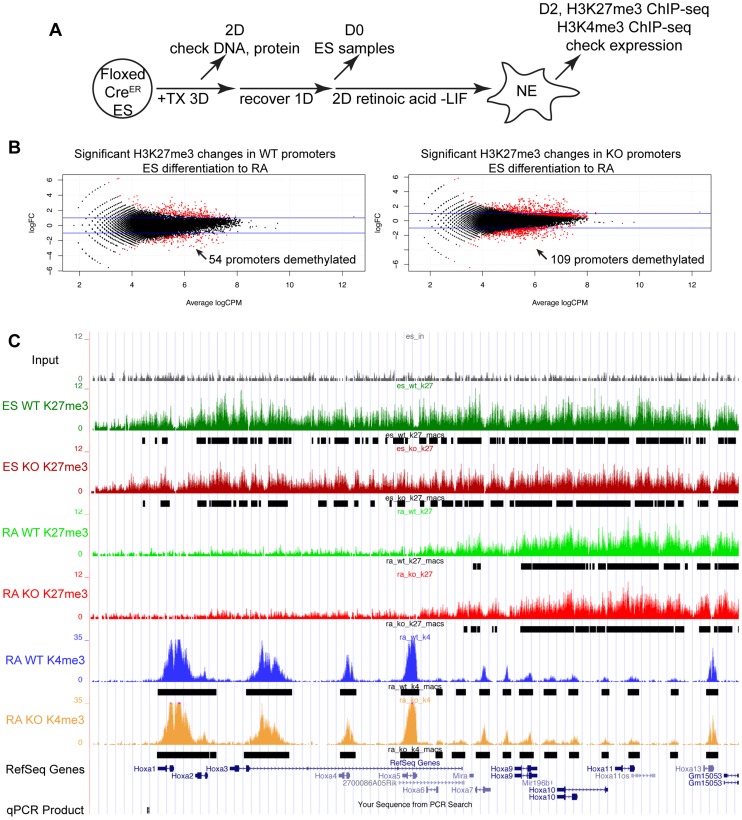
Proximal Hox genes demonstrate loss of H3K27me3 with RA treatment in the absence of KDM6 demethylation. (A) *Utx^fl/fl^*;*Jmjd3^fl/fl^*;*Cre^ER^* ES cells were untreated (WT) or pre-treated with TX for 3 days (KO), recovered, and differentiated to neuro-ectoderm with 2 days of retinoic acid treatment. H3K27me3 and H3K4me3 ChIP-seq were performed on D0 ES cells and D2 RA treated cells. (B) The normalized sequence reads from all promoters (+/−1 KB KB) from ES and RA treated cells were compared by edgeR to identify promoters that exhibit H3K27me3 reductions in either WT (*Utx^fl/fl^*;*Jmjd3^fl/fl^*;*Cre^ER^* −TX) or KO (*Utx^fl/fl^*;*Jmjd3^fl/fl^*;*Cre^ER^* +TX) cells. The log fold change (logFC) is plotted against the average log counts per million reads (Average logCPM). In the plot, 54 WT and 109 KO promoters exhibited H3K27me3 reductions with RA treatment (negative logFC, FDR<0.05, and an identified H3K27me3 MACS peak in ES cells). (C) UCSC genome browser view of ChIP-seq tracks for the *Hoxa* cluster. Illustrated are Input (black), WT ES H3K27me3 ChIP (dark green), KO ES H3K27me3 ChIP (dark red), WT RA H3K27me3 ChIP (light green), KO RA H3K27me3 ChIP (light red), WT RA H3K4me3 ChIP (blue), KO RA H3K4me3 ChIP (orange), and MACS defined enrichment peaks are illustrated as black bars underneath each track. The RARE region tested by ChIP-qPCR is noted on the bottom.

We induced embryoid body (EB) differentiation as outlined in Figure 2A. By 4 days in culture, *Utx^fl/y^*;*Jmjd3^fl/fl^*;*Cre^ER^* +TX EBs looked identical to untreated controls (Figure 2B). *Utx^fl/fl^*;*Jmjd3^fl/fl^*;*Cre^ER^* female EBs +TX were small and displayed a disorganized outer endodermal layer with cells protruding or sloughing off of the EB (Figure 2B). In contrast to aggregate EB differentiation, hanging drop EB differentiation utilized smaller starting ES cell numbers in a defined drop volume, but still produced similar EB phenotypes (Figure S5A). Embryoid bodies exhibit a characteristic decrease in global H3K27me3 levels as differentiation progresses [32], [42]. Histones were extracted from EBs to determine if this process occurs in the absence of UTX and JMJD3 demethylation. Relative to global levels of H3K27me3 in ES cells, all EBs, even *Utx^fl/fl^*;*Jmjd3^fl/fl^*;*Cre^ER^* female EBs +TX demonstrated a reduction in H3K27me3 (Figure 3C). Fluorescent quantitative western blotting verified that both male and female +TX EBs exhibit loss of H3K27me3 levels (Figure S5B,C). Therefore, early EB differentiation events coincide with downregulation of H3K27me3 levels in the absence of all KDM6.

RT-PCR across deleted exons verified that even after 8 days in culture, wild type *Utx* and *Jmjd3* expression was absent, and *Uty* expression was not diminished in TX treated cells (Figure 2D). Male *Utx^fl/y^*;*Jmjd3^fl/fl^*;*Cre^ER^* EBs +TX demonstrated normal activation of primitive ectoderm (*Fgf5* expression), mesoderm (*Flk1*), and endoderm (*Gata6*, Figure 2E). In contrast, *Utx^fl/fl^*;*Jmjd3^fl/fl^*;*Cre^ER^* female EBs +TX initiated differentiation and induced primitive ectoderm (*Fgf5*), but failed to specify meso-endoderm (Brachyury *T*, illustrated as *T*), mesoderm (*Flk1*), and endoderm (*Gata6*, Figure 2E). *Utx^fl/y^*;*Jmjd3^fl/fl^*;*Cre^ER^* ES +TX were plated to derive single cell colonies of mutant clones, and constitutive propagation of this mutant ES line over several weeks did not affect the ability of the cells to differentiate into EBs (Figure S5D,E). Overall, the severe deficits of EB differentiation in *Utx^fl/fl^*;*Jmjd3^fl/fl^*;*Cre^ER^* +TX cells does not recapitulate the mild phenotypes of *Utx^Δ/Δ^*;*Jmjd3^Δ/Δ^* female embryos (Figure 1E″).

### ES cell H3K27me3 removal and gene activation does not require KDM6 demethylases

To study the role of KDM6 in H3K27me3 demethylation, we utilized Retinoic acid (+RA) differentiation of ES cells. As *Utx^fl/fl^*;*Jmjd3^fl/fl^*;*Cre^ER^* EB +TX appeared capable of initiating ectoderm specification, RA differentiation towards a neuro-ectodermal lineage can be studied in this cellular model whereby all active demethylation by KMD6 members has been removed. We utilized a 2 day RA differentiation timecourse outlined in Figure 3A. H3K27me3 ChIP was performed on 4 individual replicates of WT ES (*Utx^fl/fl^*;*Jmjd3^fl/fl^*;*Cre^ER^* ES −TX), KO ES (*Utx^fl/fl^*;*Jmjd3^fl/fl^*;*Cre^ER^* ES +TX), WT RA (*Utx^fl/fl^*;*Jmjd3^fl/fl^*;*Cre^ER^* RA −TX), and KO RA (*Utx^fl/fl^*;*Jmjd3^fl/fl^*;*Cre^ER^* RA +TX). Two of the 4 replicates of each group were pooled together and the resulting 2 replicates of each group were subject to high throughput sequencing. H3K4me3 ChIP-seq was also performed on 2 replicates of WT RA and KO RA. The model-based analysis for ChIP-seq (MACS) algorithm identified enrichment peaks of H3K27me3 and H3K4me3 in each group, and edgeR statistical analysis software identified genes undergoing H3K27me3 demethylation in WT (WT ES vs. WT RA) and KO (KO ES vs. KO RA) RA differentiation. Overall, 1044 WT ES promoters (Transcription Start Site: TSS +/−1 KB) and 1141 KO ES promoters demonstrated H3K27me3 peaks. Of these promoters, 945 and 1055 (WT and KO respectively) also had a RA H3K4me3 peak, signifying that the majority of these promoters are bivalent.

**Figure 4:**
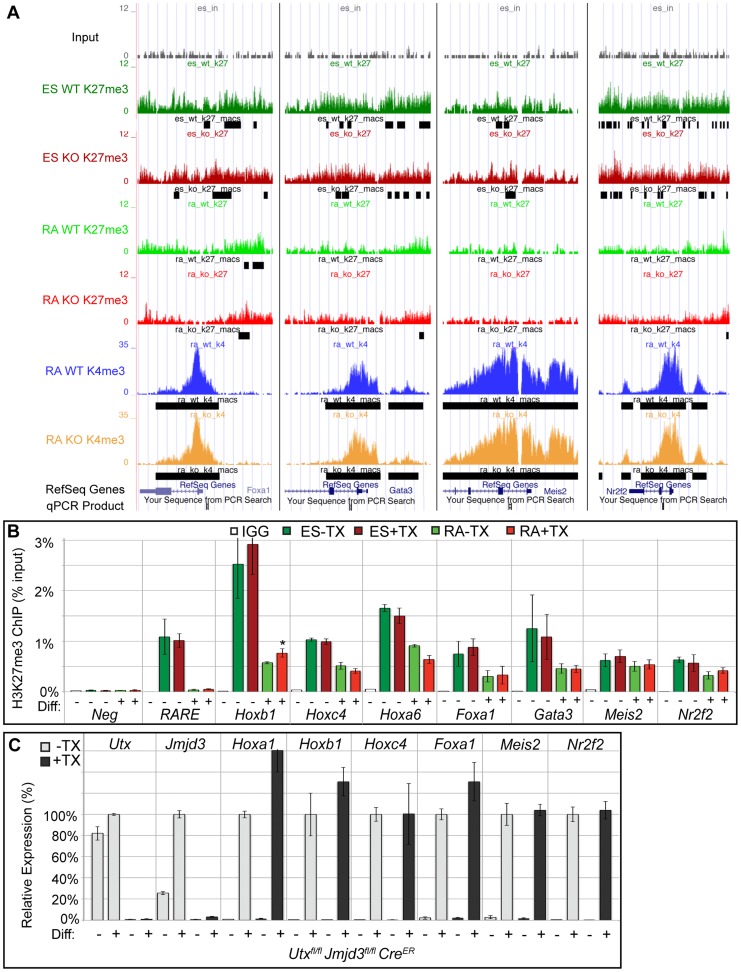
Several developmental genes demonstrate loss of H3K27me3 with RA treatment in the absence of KDM6 demethylation. (A) UCSC genome browser view of *Foxa1*, *Gata3*, *Meis2*, and *Nr2f2*. Illustrated are Input (black), WT ES H3K27me3 ChIP (dark green), KO ES H3K27me3 ChIP (dark red), WT RA H3K27me3 ChIP (light green), KO RA H3K27me3 ChIP (light red), WT RA H3K4me3 ChIP (blue), KO RA H3K4me3 ChIP (orange), and MACS defined enrichment peaks are illustrated as black bars underneath each track. Regions tested by ChIP-qPCR are noted on the bottom. (B) Verification of H3K27me3 loss by ChIP-qPCR. H3K27me3 ChIP of *Utx^fl/fl^*;*Jmjd3^fl/fl^*;*Cre^ER^* ES cells (dark green and red, Diff −) or after 2 days of RA treatment (light green or red, Diff +) left untreated (green) or pre-treated with tamoxifen (red). An IgG control ChIP is illustrated as white bars. Quantitative PCR of a H3K27me3 negative locus (*Slc2a8* promoter, *Neg*) was utilized for comparison to the *Hox* A cluster retinoic acid response element (*RARE*), *Hoxb1*, *Hoxc4*, *Hoxa6*, *Foxa1*, *Gata3*, *Meis2*, and *Nr2f2* promoters. N = 4 samples per treatment. All genes tested exhibited demethylation in both WT and KO cells, with only Hoxb1 demonstrating a slight, but significant increase in KO RA treatment (p-value = 0.03). (C) Quantitative RT-PCR of *Utx*, *Jmjd3*, and indicated *Hox* genes, *Foxa1*, *Meis2*, and *Nr2f2* from *Utx^fl/fl^*;*Jmjd3^fl/fl^*;*Cre^ER^* ES cells (Diff −) or after 2 days of RA treatment (Diff +) left untreated (−TX, light grey) or pre-treated with tamoxifen (+TX, black). N = 3 samples per treatment. All samples are normalized relative to −TX Differentiation + RA treatment. No genes tested demonstrated significantly reduced expression in KO RA cells.

EdgeR identified 54 WT promoters (from 50 unique genes, some having alternative promoters) and 109 KO promoters (103 genes) that demonstrated significant loss of H3K27me3 with RA differentiation (Figure 3B and Table S1) with many genes overlapping in both datasets. Many *Hox* genes lost H3K27me3 in both WT and KO RA differentiation. Tracks of H3K27me3 and H3K4me3 ChIP-seq were uploaded into the UCSC genome browser. With RA differentiation, the proximal *Hoxa* cluster (Figure 3C) from *Hoxa1* through *Hoxa6* demonstrated widespread loss of H3K27me3 in ES to RA differentiation of both WT and KO cells. This shift in histone profile correlated with large peaks of H3K4me3 at proximal *Hoxa* promoters, demonstrating gene activation events. Similar large-scale loss of H3K27me3 occurs from the proximal *Hoxb* (*Hoxb1-Hoxb6*), *Hoxc* (*Hoxc4-Hoxc6*), *and Hoxd* (*Hoxd1-Hoxd4*) clusters (Figure S6A,B,C). In addition to *Hox* genes, many other transcription factors such as *Foxa1*, *Gata3*, *Meis2*, and *Nr2f2* demonstrated loss of promoter H3K27me3 in KO RA treatment (Figure 4A and Table S1). H3K27me3 ChIP-qPCR confirmed the loss of H3K27me3 in the absence of KDM6 demethylation (Figure 4B). Notably, all genes tested exhibited similar WT and KO loss of H3K27me3 with RA differentiation. Only *Hoxb1* demonstrated a slight, significant increase in H3K27me3 for KO RA compared to WT RA, but overall *Hoxb1* did exhibit a tremendous decrease in H3K27me3 in KO RA compared to KO ES. Furthermore, RT-PCR expression analysis confirmed that the genes demonstrating loss of H3K27me3 efficiently induced transcriptional activation in RA KO cells (Figure 4C). *Utx^fl/fl^*;*Jmjd3^fl/fl^*;*Cre^ER^* ES +TX were plated to derive single cell colonies of mutant clones, and constitutive propagation of this mutant ES line over several weeks did not affect the ability of the cells to activate transcription (Figure S5F). H3K27 demethylases physically associate with the MLL complex family of H3K4 methyl-transferases. Two members of this complex (ASH2L and RBBP5) were expressed at normal levels in KO cells (Figure S7A). One alternative explanation for loss of H3K27me3 in the absence of demethylation is that the PRC2 H3K27 methylation complex is down-regulated in differentiated cells or displaced from targeted promoters in a demethylase independent manner. While the EZH2 H3K27 methyl-transferase maintained high expression in RA differentiated cells (Figure S7B), the protein is displaced from promoters experiencing H3K27me3 loss in the absence of KDM6 demethylation (). In summary, repressed genes can demonstrate loss of H3K27me3 and initiate expression in the absence of KDM6.

### Loss of KDM6 reduces H3K4 methylation and transcriptional activation in a small subset of H3K4me3 regulated genes

**Figure 5:**
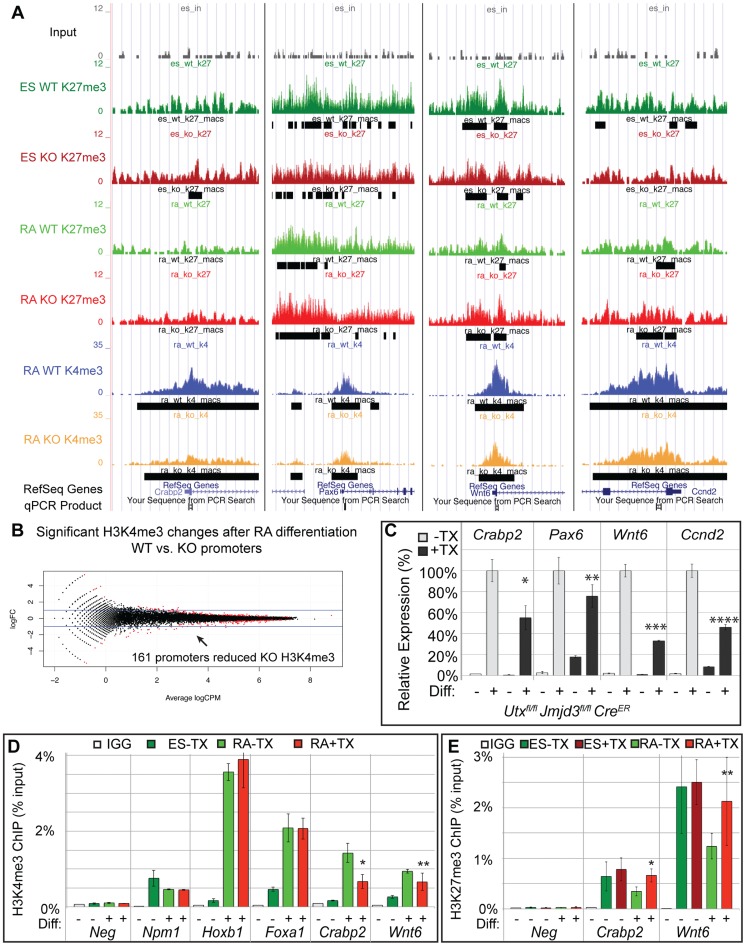
A subset of genes exhibit reduction of H3K4me3 with loss of UTX and JMJD3. (A) UCSC genome browser view of *Crabp2*, *Pax6*, *Wnt6*, and *Ccnd2*. Illustrated are Input (black), WT ES H3K27me3 ChIP (dark green), KO ES H3K27me3 ChIP (dark red), WT RA H3K27me3 ChIP (light green), KO RA H3K27me3 ChIP (light red), WT RA H3K4me3 ChIP (blue), KO RA H3K4me3 ChIP (orange), and MACS defined enrichment peaks are illustrated as black bars underneath each track. Regions tested by ChIP-qPCR are noted on the bottom. (B) H3K4me3 ChIP-seq was performed on WT (*Utx^fl/fl^*;*Jmjd3^fl/fl^*;*Cre^ER^* −TX) or KO (*Utx^fl/fl^*;*Jmjd3^fl/fl^*;*Cre^ER^* +TX) cells treated with RA. The normalized sequence reads from all promoters (+/−1 KB KB) from RA treated cells were compared by edgeR to identify promoters that exhibit H3K4me3 reductions in KO cells. The log fold change (logFC) is plotted against the average log counts per million reads (Average logCPM). In the plot, 161 KO promoters exhibited H3K27me3 reductions with RA treatment (negative logFC, FDR<0.05, and an identified H3K4me3 MACS peak in WT RA cells). (C) Quantitative RT-PCR of *Crabp2*, *Pax6*, *Wnt6*, and *Ccnd2* from *Utx^fl/fl^*;*Jmjd3^fl/fl^*;*Cre^ER^* ES cells (Diff −) or after 2 days of RA treatment (Diff +) left untreated (−TX, light grey) or pre-treated with tamoxifen (+TX, black). N = 3 samples per treatment. All samples are normalized relative to −TX Differentiation + RA treatment. Significantly reduced expression is demonstrated (p-vale = *0.01, **0.03, ***0.001, ****0.004). (D) Verification of H3K4me3 reductions in KO RA cells by ChIP-qPCR. H3K4me3 ChIP of *Utx^fl/fl^*;*Jmjd3^fl/fl^*;*Cre^ER^* ES cells (dark green, Diff −) or after 2 days of RA treatment (light green or red, Diff +) left untreated (green) or pre-treated with tamoxifen (red). An IgG control ChIP is illustrated as white bars. Quantitative PCR of a H3K4me3 negative locus (gene desert region, *Neg*) was utilized for comparison to *Npm1* (a positive control), genes exhibiting normal KO gene activation (*Hoxb1, Foxa1*), and genes with reductions in KO H3K4me3 (*Crabp2, Wnt6*). Only *Crabp2* and *Wnt6* demonstrated decreases in KO RA treatment (p-value = *0.01, **0.07). (E) H3K27me3 ChIP of *Crabp2* and *Wnt6* relative to a negative control (Slc2a8). Both genes demonstrated increases in H3K27me3 in RA KO cells (light red bars, p-value = *0.003, **0.03).

MACs analysis of H3K4me3 ChIP-seq of WT RA and KO RA cells identified enrichment peaks at 19,140 and 19,367 respective promoters (TSS +/−1 KB). EdgeR comparison of WT RA to KO RA identified 161 promoters (147 genes) that exhibited significant (FDR<0.05) reduction in KO H3K4me3 (Figure 5B, Table S2). The majority of these were small fold changes across a wide range in overall peak intensity. Of these 161 promoters, only 27 (26 genes) had an ES WT or KO H3K27me3 peak, so the majority of these genes are not regulated by H3K27 methylation. A few genes that exhibited KO H3K4me3 reductions and had a H3K27me3 peak are illustrated in the UCSC genome browser image of Figure 5A. RT-PCR confirmed that several genes with reduced KO H3K4me3 demonstrated reduced expression (*Crabp2*, *Pax6*, *Wnt6*, *Ccnd2*, Figure 5C). Several genes that had been denoted as experiencing normal H3K27me3 loss and gene activation in KO RA samples (*Hoxb1*, *Foxa1*) experienced normal KO RA H3K4me3 up-regulation relative to the ES cell state (Figure 5D). However, ChIP-qPCR did confirm genes identified in ChIP-seq to be deficient in RA KO H3K4me3 (*Crabp2*, *Wnt6*, Figure 5D). We next examined whether KO RA H3K4me3 affected genes can have associated increases in H3K27me3. ChIP-qPCR of *Crabp2* and *Wnt6* demonstrated increased KO RA H3K27me3 to ES comparable levels (Figure 5E).

### Meta-analysis of categorized gene subsets reveals low-level changes in H3K27me3 in the absence of KDM6 demethylation

**Figure 6:**
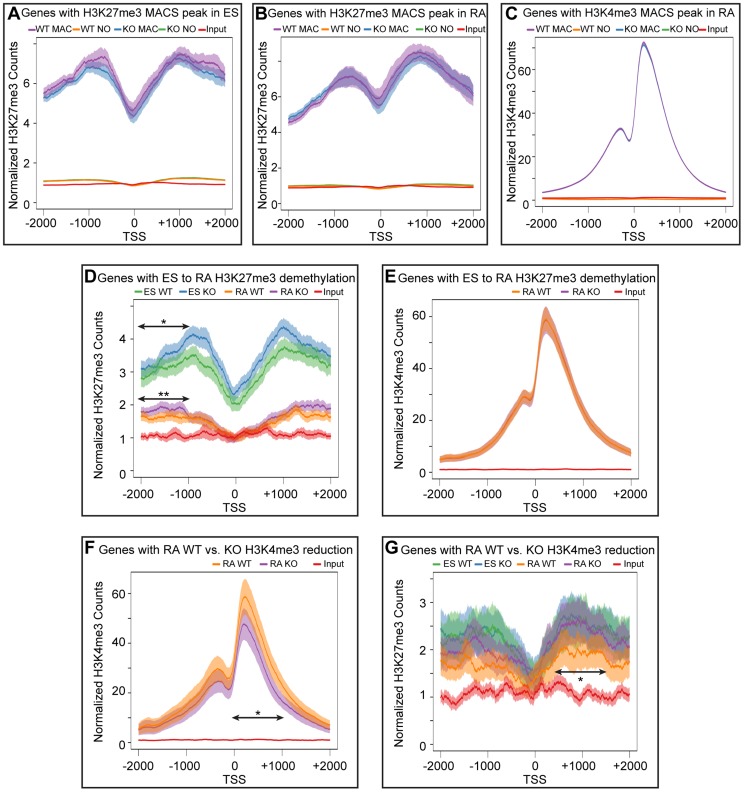
Meta-analysis of H3K27me3 and H3K4me3 distribution across female KDM6 WT and KO promoters. (A) Meta-analysis plotted the normalized H3K27me3 sequence reads and corresponding 95% confidence interval for all female KDM6 WT and KO ES promoters with a H3K27me3 MACS peak against all promoters without a MACS peak. Gene distributions span −2000 base pairs upstream of the transcription start site (TSS) to +2000 downstream. (B) Normalized H3K27me3 sequence reads for all WT and KO RA promoters with a H3K27me3 MACS peak against all promoters without a MACS peak. (C) Normalized H3K4me3 sequence reads for all WT and KO RA promoters with a H3K4me3 MACS peak against all promoters without a MACS peak. (D) Normalized H3K27me3 sequence reads across promoters identified by edgeR to experience H3K27me3 loss with RA differentiation. ES WT and KO vs. RA WT and KO are illustrated. While there is a large drop-off in both WT and KO RA counts, KO ES and KO RA was slightly elevated relative to WT ES and WT RA respectively across −1000 to −2000 (Ttest of means for all given promoters, p-value = *0.036, **0.019) (E) Normalized H3K4me3 sequence reads across promoters identified by edgeR to experience H3K27me3 loss with RA differentiation. RA WT and KO are illustrated. (F) Normalized H3K4me3 sequence reads across promoters identified by edgeR to experience H3K4me3 loss in KO RA relative to WT RA. RA WT and KO are illustrated. KO RA is significantly reduced across 0 to +1000 (p-value = *0.009). (G) Normalized H3K27me3 sequence reads across promoters identified by edgeR to experience H3K4me3 loss in KO RA relative to WT RA. ES WT and KO vs. RA WT and KO are illustrated. KO RA was significanly elevated relative to WT RA across +500 to +1500 (p-value = *0.040).

To better analyze the distribution of H3K27me3 and K3K4me3 we performed a meta-analysis examining the overall distribution of these histone modifications across all promoters with a corresponding MACS peak. These meta analyses (Figure 6A–G) were plotted with 95% confidence intervals centered on the mean normalized read counts. ES cells and RA differentiated cells both had a broad K27me3 distribution across promoters with a drop-off near the TSS (Figure 6A,B). H3K4me3 enrichment peaked downstream of the TSS (Figure 6C), and there was very close overlap between WT and KO profiles of both H3K27me3 and H3K4me3. Genes with significant reductions in H3K27me3 after RA differentiation, had visible differences in relative H3K27me3 sequence reads between ES and RA samples for both WT and KO (Figure 6D). In comparing WT RA to KO RA, there was very close overlap near the TSS (+/−1 KB); however, upstream of the TSS (−2 KB to −1 KB) KO RA exhibited a significant enrichment in the mean H3K27me3 levels per gene. Comparison of WT ES to KO ES across this same region also demonstrated significantly increased H3K27me3 levels (Figure 6D). Although H3K27me3 was elevated for these KO promoters, there was a complete overlap in H3K4me3 distribution (Figure 6E), further supporting data that this gene category was efficiently activating transcription in KO RA cells (Figure 4C). Thus, with RA differentiation, KO cells can experience dramatic reductions in H3K27me3 at specific promoters, and although there is minor H3K27me3 accumulation upstream of the TSS, transcription is not compromised.

Meta-analysis of promoters exhibiting H3K4me3 reductions in KO RA cells verified that this dataset was significantly deficient in H3K4me3 downstream of the TSS (Figure 6F). The H3K27me3 profile of this dataset revealed that these genes were not subject to dramatic K3K27me3 loss in WT ES to WT RA differentiation (Figure 6G). However, there was a small, but significant H3K27me3 elevation in RA KO relative to RA WT from TSS +0.5 KB to +1.5 KB. Because identifying a small subset of data from a graph for statistical analysis amounts to cherry-picking and is not without bias, we performed a genome-wide comparison of WT RA to KO RA H3K27me3 levels with edgeR. Genome-wide cross-comparison of WT RA vs. KO RA did not identify any promoters (TSS +/−1 KB) exhibiting a significant increase in KO H3K27me3. We also compared all KO RA H3K27me3 MACS peaks (peak center +/−0.5 KB), including those not found at TSSs, for normalized sequence read enrichment over WT RA. This analysis identified 74 KO RA H3K27me3 MACS peaks (out of 4504 total MACS peaks) that were enriched in sequence reads over WT (Table S3). These peaks resided in varying proximity to 64 unique genes; however, only 7 of these genes demonstrated compromised transcription based on a reduction in TSS H3K4me3 levels (Table S3). Overall, with KDM6 loss of demethylation, a small subset of genes have minor reductions in H3K4me3 and reduced transcription, and a fraction of these experience elevated H3K27me3.

### MEFs exhibit KDM6 independent loss of H3K27me3 from promoters

**Figure 7:**
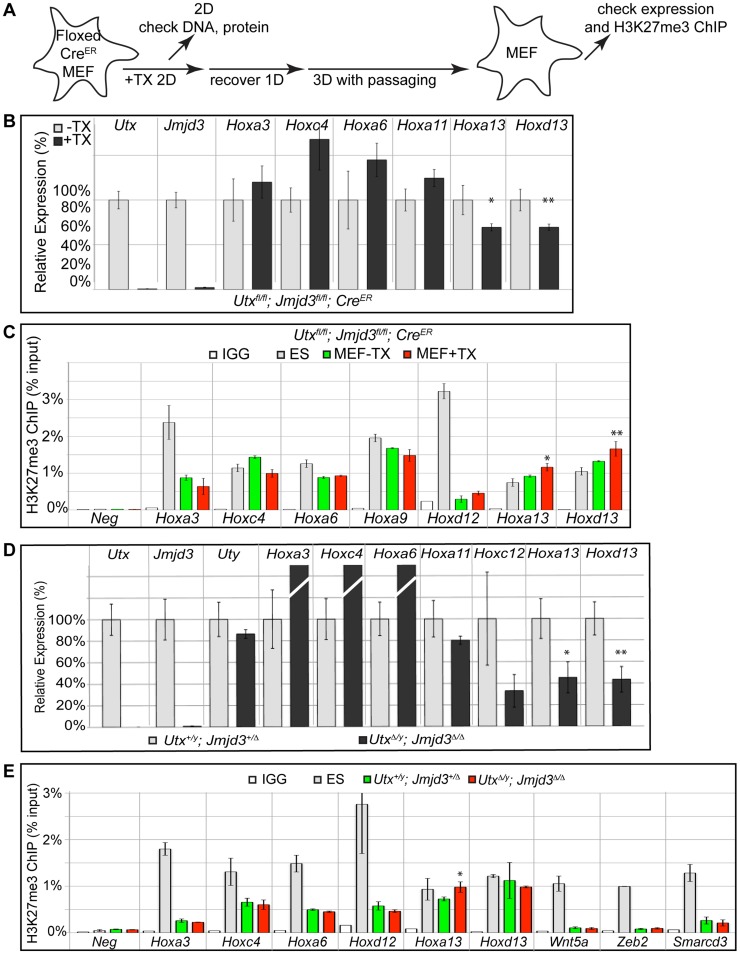
RT-PCR and H3K27me3 ChIP of *Utx and Jmjd3* mutant MEFs. (A) Schematic of MEF TX treatment. (B) Quantitative RT-PCR of indicated genes from *Utx^fl/fl^*;*Jmjd3^fl/fl^*;*Cre^ER^* mouse embryonic fibroblasts (MEFs) left untreated (−TX, light grey) or pre-treated with TX (+TX, black). Significant reductions in *Hox* expression are indicated (T-test p-value * = 0.05, ** = 0.02, N = 3 samples per treatment). (C) H3K27me3 ChIP of *Utx^fl/fl^*;*Jmjd3^fl/fl^*;*Cre^ER^* MEFs left untreated (green) or pre-treated with TX (red). An IgG control ChIP is illustrated as white bars and H3K27me3 ChIP of ES cells is illustrated as light grey bars. Quantitative PCR of a H3K27me3 negative locus (*Slc2a8* promoter, *Neg*) was utilized for comparison to *Hox* promoters. Significant H3K27me3 accumulations of MEF +TX relative to −TX are indicated (T-test p-value * = 0.01, ** = 0.04, N = 3 samples per treatment). (D) Quantitative RT-PCR of indicated genes from *Utx^+/y^*;*Jmjd3^+/Δ^* (light grey bars) or *Utx^Δ/y^*;*Jmjd3^Δ/Δ^* MEFs (black bars). Significant reductions in *Hox* expression are indicated (T-test p-value * = 0.01, ** = 0.003, N = 3 independent MEF lines per treatment). (E) H3K27me3 ChIP of *Utx^+/y^*;*Jmjd3^+/Δ^* (green bars) or *Utx^Δ/y^*;*Jmjd3^Δ/Δ^* MEFs (red bars). An IgG control ChIP is illustrated as white bars and H3K27me3 ChIP of ES cells is illustrated as light grey bars. Quantitative PCR of a H3K27me3 negative locus (*Slc2a8* promoter, *Neg*) was utilized for comparison to promoters of *Hox* genes and other indicated transcription factors. Significant H3K27me3 accumulations of *Utx^Δ/y^*;*Jmjd3^Δ/Δ^* MEFs relative to *Utx^+/y^*;*Jmjd3^+/Δ^* MEFs are indicated (T-test p-value * = 0.02, N = 3 samples per treatment).

To examine loss of H3K27me3 repression in a differentiated primary tissue, we utilized mouse embryonic fibroblasts (MEFs). Primary MEFs were cultured from E13.5 *Utx^fl/fl^*;*Jmjd3^fl/fl^*;*Cre^ER^* embryos and treated with tamoxifen as indicated in Figure 7A. Relative to ES cells, a panel of representative *Hox* genes (*Hoxa3*, *Hoxc4*, *Hoxa13* and *Hoxd13*) demonstrated elevated expression levels in MEFs (Figure S8A). Following tamoxifen treatment, the growth of *Utx^fl/fl^*;*Jmjd3^fl/fl^*;*Cre^ER^* MEFs slowed dramatically, while MEFs without Cre continued to proliferate. Regardless, *Utx^fl/fl^*;*Jmjd3^fl/fl^*;*Cre^ER^* MEFs +TX largely did not experience reductions in *Hox* expression relative to untreated controls (Figure 7B). Only the most distal genes within the *Hox* A and D clusters (*Hoxa13* and *Hoxd13*) demonstrated a slight but significant reduction in expression with loss of UTX and JMJD3. *Hox* H3K27 methylation in *Utx^fl/fl^*;*Jmjd3^fl/fl^*;*Cre^ER^* MEFs +TX matched their expression profile as the few distal genes that demonstrated mild expression deficiencies (*Hoxa13* and *Hoxd13*) also exhibited a significant increase in H3K27me3 (Figure 7C).

Primary MEFs were also cultured from E13.5 *Utx^Δ/y^*;*Jmjd3^Δ/Δ^* embryos to assay function in both establishment and maintenance of a H3K27 demethylated state. Similar to transient TX induced KDM6 loss, *Utx^Δ/y^*;*Jmjd3^Δ/Δ^* MEFs had significantly reduced expression of more distal *Hox* genes (*Hoxa13*, *Hoxd13*, Figure 7D) relative to *Utx^+/y^*;*Jmjd3^+/Δ^* controls. H3K27me3 ChIP of *Utx^Δ/y^*;*Jmjd3^Δ/Δ^* MEFs only revealed some accumulation on *Hoxa13* while other *Hox* genes were unaffected (Figure 7E). Notably, several *Hox* genes demonstrated significant reduction in H3K27me3 levels relative to ES cells (*Hoxa3*, *Hoxc4*, *Hoxa6*, *Hoxd12*) in both control and *Utx^Δ/y^*;*Jmjd3^Δ/Δ^* MEFs (Figure 7E). Some proximal Hox genes actually demonstrated an increase in KO MEF *Hox* expression (*Hoxa3*, *Hoxc4* and *Hoxa6*), but this may be an artifact of the decreased growth rate of these cells as the H3K27me3 profile of these genes is unaffected. Furthermore, several other transcription factors (*Wnt5a*, *Zeb2*, *Smarcd3*) also exhibited near-complete loss of H3K27me3 even though all KDM6 demethylases had been removed throughout embryonic development. Notably these genes experience loss of localized H3K27me3 even though total H3K27me3 protein levels (Figure S3G,H) and EZH2 levels (Figure S8B,C) were elevated (H3K4me3 levels were not affected, Figure S8C). Therefore, H3K27me3 repressed genes can establish promoter states cleared of this repressive chromatin in the absence of KDM6 demethylases.

## Discussion

A tremendous dichotomy exists in the field of H3K27 demethylases. H3K27me3 results in gene repression throughout the early embryo, yet enzymes that catalyze its removal are individually not essential for male embryonic viability. These findings are unexpected given that numerous genes crucial for early embryonic gastrulation events [43]–[52] experience H3K27me3 de-repression during development [4], [53], [54] including but not limited to GATA, TGF-β/BMP, WNT, FGF, and T-box transcription factor networks. Cell culture model systems have implicated that UTX and JMJD3 function in activation of several of these pathways [17]–[19], [22], [32]. We now demonstrate *Utx^Δ/y^*;*Jmjd3^Δ/Δ^* embryos devoid of all KDM6 demethylation remarkably survive to term and appear phenotypically normal at mid-gestation. *Utx^Δ/Δ^*;*Jmjd3^Δ/Δ^* embryos (lacking the demethylase independent function of UTY) survive through gastrulation and albeit smaller in size, can develop E10.5 features. Therefore, KDM6 is not crucial for alleviating the H3K27me3 repression of genes needed for early embryonic gastrulation events.

Even within individual cellular and organismal models, several studies have produced conflicting reports. UTX mediated H3K27 demethylation is reported to function in cellular reprogramming and germ cell development [55], however surviving *Utx* mutant male mice are fertile [31], [56]. UTX is reported to be essential for appropriate expression of germ layer markers in male ES cell differentiation [19], [56], yet differentiation deficits can largely be rescued by UTY or a catalytically inactive form of UTX [32], [57]. UTX is essential for efficient *Hox* H3K27me3 demethylation and gene activation [27], [29], [30], [57], [58], yet Utx null male ES cells can largely remove *Hox* H3K27me3 and demonstrate normal transcriptional activation [56]. The discrepancies in these reports may be accounted for by differences in cell type, genetic background, intrinsic growth differences in ES cell clones, differential JMJD3 redundancy, or differential UTY expression [as has been reported [31], [32], [56], [59]. We take advantage of mutant inducible alleles to enable comparison of control and knockout of the entire KDM6 family within the same ES cell clone with identical growth rate and genetic background. Similar to the normal appearance of mid-gestation *Utx^Δ/y^*;*Jmjd3^Δ/Δ^* embryos, KDM6 mutant male ES cells could differentiate normally into all EB germ layers. Mutant female ES cells null for any KDM6 demethylation demonstrated dramatic reduction in both global levels of H3K27me3 (with EB differentiation) and local levels of H3K27me3 from proximal *Hox* clusters and from promoters of other transcription factors (with RA differentiation). While there was a low level significant H3K27me3 accumulation upstream of these promoters in RA KO cells, these genes were largely cleared of H3K27me3 and experienced normal transcriptional activation, similar to findings in *Utx* male knockout studies alone [56]. *Utx* and *Jmjd3* mutant MEFs demonstrated mild H3K27me3 accumulation and reduced expression only within the most distal regions of the *Hox* cluster. Similarly, Zebrafish UTX loss of function produces modest deficiencies in distal *Hox* expression [27]. EZH2 protein levels were mildly upregulated in *Kdm6* mutant MEFs and may account for altered distal *Hox* gene regulation. Relative to ES cells, *Utx^Δ/y^*;*Jmjd3^Δ/Δ^* MEFs exhibited substantial reductions in promoter H3K27me3 of several Hox genes and developmental transcription factors. Thus in the absence of KDM6 H3K27 demethylation, H3K27me3 loss can be both initiated and maintained in developmental situations.

Our study raises intriguing questions regarding early embryonic removal of H3K27me3. Thus far, only UTX and JMJD3 have demonstrated the ability to demethylate H3K27me3. It is unlikely that another JmjC protein can demethylate H3K27me3. The KDM7 family including JHDM1D and PHF8 can demethylate both H3K9 and H3K27 dimethyl residues, but does not demethylate trimethyl residues [60], [61]. The PHF8 active site cannot sterically accommodate trimethyl residues [62]. In contrast, UTX positions H3K27me3 farther from the active site to properly position the larger residue modifications [63]. Furthermore, UTX amino acid Y1135 bonds with a methyl group of H3K27me3 and is essential for demethylation [31], [63]. This residue is not conserved in the KDM7 family. A tyrosine at this position is conserved for members of the KDM4 family of H3K9 and H3K36 trimethyl demethylases. However, this family of proteins has a catalytic core buried within a deep pocket, and residues downstream of H3K27 do not encode enough flexibility to fit this modification in the active site [64]. It is possible that a novel family of proteins may actively demethylate H3K27me3 utilizing distinct chemistry.

Alternatively, H3K27me3 in the early embryo may be replaced by passive mechanisms, as histones can be turned over multiple times within each cell cycle [65]. PRC complexes remain bound to chromatin during DNA replication and associate with the replication fork in dividing cells to direct methylation of H3K27me3 on newly incorporated daughter strand histones [66]–[68]. Thus, in a passive model for H3K27me3 replacement, displacement of the PRC2 complex during replication allows for incorporation of un-methylated H3K27. In a similar fashion, DNA methylation in the mouse pre-implantation embryo and germline may be removed via passive replication dependent mechanisms via displacement of DNA methyl-transferase from sites of replication [69], [70].

The role of the KDM6 family in development is not clear. Passive H3K27me3 removal may dominate in rapidly dividing cells such as in the early embryo. Active H3K27me3 demethylation may prove more essential for rapid response to specific environmental or developmental cues, particularly in more static cellular populations. Alternatively, rather than facilitating drastic gene induction, H3K27 demethylases may act to fine-tune transcriptional activity to promote accurate robust temporal and spatial patterns of gene expression. Accordingly, UTY associates with a wide array of chromatin and transcriptional machinery that may promote proper gene expression output [31]. In this sense, KDM6 members may encode a reader function to recruit transcriptional complexes to repressed genes and/or to displace PRC2 without the need for active demethylation. Point mutagenesis in the UTX and JMJD3 catalytic domains further emphasizes demethylase independent roles for the KDM6 family [32], [71], [72]. Going forward, genetic experiments analyzing crosstalk between chromatin modifying factors and structure/function analysis within the mammalian embryo are required to define specific function of the KDM6 family in embryonic development.

## Materials and Methods

### Mice

All mouse experimental procedures were approved by the University of North Carolina Institutional Animal Care and Use Committee. The *Utx^fl^* allele is described [31]. The *Jmjd3^tm1Mag^* (*Jmjd3^fl^*) allele was derived by targeting E14 ES cells. The *Jmjd3* targeting construct was generated by BAC recombineering to insert a LoxP site in intron 13 and a FRT-Neomycin-FRT-LoxP cassette in intron 20 of *Jmjd3*. After successful targeting, the Neomycin cassette was removed by electroporation of a pCAGG-FLP construct. The resulting *Jmjd3^+/fl^* ES cells were injected into C57BL/6J blastocysts. Resulting chimeras were mated to CD1 females to assess germline transmission, and were maintained on either a mixed CD1 background or were backcrossed to C57BL/6J. *Utx^Δ^* and *Jmjd3^Δ^* alleles were generated by crossing floxed alleles to the *VasaCre* transgene that restricts Cre activity to the germline [73]. These progeny were then mated to propagate the *Utx^Δ^* and *Jmjd3^Δ^* alleles. To generate higher proportions of desired genotypes in the *Utx Jmjd3* genetic interaction cross, *Utx^+/Δ^;Jmjd3^+/Δ^* female mice were mated with either *Utx^fl/y^;Jmjd3^fl/Δ^;VasaCre* or *Utx^+/y^;Jmjd3^fl/Δ^;VasaCre* males. Embryos were PCR genotyped from yolk sac samples for *Utx* or *Jmjd3* and were sexed by a PCR genotyping scheme to distinguish *Utx* from *Uty*. All primer sequences are listed in Table S4.

### Cell culture

*Utx^fl/fl^*;*Jmjd3^fl/fl^*;*Cre^ER^* and *Utx^fl/y^*;*Jmjd3^fl/fl^*;*Cre^ER^* ES cell lines were generated from E3.5 blastocysts in crosses utilizing *CAGGCre-ER* transgenic mouse line [41]. ES cells were cultured as described [36]. ES cells were split off of feeder MEFs and treated with 1 µg/mL 4-hydroxytamoxifen (4-OHT) for 3 days and were allowed to recover for 1 day. In EB differentiation experiments, 10^6^ ES cells were cultured in 10 mL of ES culture media lacking LIF on agarose coated Petri dishes. Hanging drop EBs were set up with 25 µL drops of ES cells at a concentration of 20,000 cells per mL. In RA differentiation experiments, ES cells were plated following tamoxifen recovery, and the next day were cultured without LIF in 1 µM Retinoic Acid. E13.5 MEFs were generated as described [31]. MEFs were passaged 1x and treated with 0.5 µM 4-OHT for 2 days, then were passaged and cultured for an additional 3 days.

### RT-PCR, western blotting, and ChIP

RNA was isolated with Trizol and cDNA was synthesized with Multiscribe reverse transcriptase. Gene expression was analyzed by qRT-PCR (Bio-Rad SsoFast EvaGreen, CFX96 real time system). All RT-PCR was normalized to *Gapdh* expression and graphed relative to control samples. All primer sequences are listed in Table S4. Nuclear lysates, histone extracts, and western blotting was performed as described [31] utilizing anti-RBBP5 (Bethyl Labs A300-109A, 1∶5000), anti-ASH2L (Bethyl Labs A300-107A, 1∶3000), anti-H3K27me3 (Millipore 07-449, 1∶2000), anti-H3 (Abcam ab1791, 1∶10,000), anti-GAPDH (Sigma G9545, 1∶10000), anti-UTX [29], or anti-UTX (Bethyl Labs A302-374A, 1∶4000) antibodies. ChIP was performed as described [74] and graphed relative to % of total ChIP input DNA for each immunoprecipitation. 5×10^6^ cells were sonicated by a Branson Sonifier at 15% duty cycle (0.7 s on 0.3 s off). For some experiments, chromatin was sonicated in a chilled water bath by a Bioruptor sonifier on high setting for 30 s with 60 s rest. Rabbit IgG (Sigma, I5006), anti-H3K27me3 (Abcam ab6002, 2.5 µl), anti-H3K4me3 (Abcam ab8580, 2 µl), or EZH2 (Cell Signaling 5246, 3 µl) antibodies were used for ChIP. All ChIP primers are listed in Table S4. Immunofluorescence experiments were performed as described [31] with anti H3K27me3 (Cell Signaling 9733S, 1∶500) or ISL1 (DSHB 39.4D5-S, 1∶200)

### Preparation of ChIP-seq libraries and data analysis

ChIP DNA and Input DNA were ligated to Truseq adapters as described [75]. Samples were multiplexed and sequenced with the HiSeq 2000 Analyzer (UNC High Throughput Sequencing Facility). The quality of the sequences reads was evaluated with FastQC (http://www.bioinformatics.babraham.ac.uk/projects/fastqc/). Reads were then mapped to the B6 genome using Bowtie (http://bowtie-bio.sourceforge.net/index.shtml). Significant enrichment (peaks) were called using MACS (http://liulab.dfci.harvard.edu/MACS/index.html), with the input ChIP-seq datasets for the background model, on pooled replicates. Peak lists were filtered using an FDR cutoff of 0.05. We then identified peaks that were within 1 Kb of transcriptional start sites (TSSs), as annotated in the UCSC genome browser for mm9. Read counts for a particular locus were normalized to the total number of sequence reads generated for each sample. We used edgeR (http://www.bioconductor.org/packages/2.12/bioc/html/edgeR.html) to determine if there was a bias in the number of H3K27me3 or H3K4me3 reads at MACS positive promoters between various samples. Metaplots were drawn using custom Python scripts and R, and t-tests were performed using the average read count per gene within the ranges specified in the text. ChIP-seq datasets were submitted to GEO (accession GSE58391: http://www.ncbi.nlm.nih.gov/geo/query/acc.cgi?acc=GSE58391).

## Supporting Information

Figure S1 Targeted mutation of *Jmjd3*. (A) *Jmjd3* was targeted to introduce a LoxP site in intron 13 and another in intron 20. The FRT site remains after the Neo cassette was removed by FLP recombinase. A schematic of the JMJD3 protein is illustrated above to denote the region that will be removed with Cre recombinase. Splicing from exon 13–21 will introduce a frameshift and stop codon. (B) Verification of the *Jmjd3^fl^* allele. Southern blotting of WT and targeted *Jmjd3^+/neofl^* ES cells with a 3′ probe and XmnI digest demonstrated the expected shift in banding due to a novel restriction site. The neo cassette was removed from targeted ES cells by transfection of a Flp recombinase to create *Jmjd3^+/fl^*. (C) *Jmjd3^fl^* ^/fl^ mice were crossed to a germline Cre recombinase to create *Jmjd3^Δ/Δ^*. A PCR genotyping scheme was designed to distinguish WT, *Jmjd3^fl^*, and *Jmjd3^Δ^* alleles in resulting mice. (D) The *Jmjd3* deletion is verified by RT-PCR of E18.5 whole embryo RNA. *Jmjd3^Δ/Δ^* embryos lack the exons 14–17 product and amplification across exons 13–22 produces a smaller band that corresponds to the expected product lacking exons 14–20. Therefore, the mutant transcript is stable and is expected to produce JMJD3 lacking the JmjC histone demethylase domain. (E) Homozygous *Jmjd3* mutant embryos are born, but fail to breathe as indicated by their blue color. (F) *Jmjd3^Δ/Δ^* embryos appear phenotypically normal at E10.5.
(TIF)

Figure S2 Maternal deletion of UTX and JMJD3 does not enhance embryonic mutant phenotypes. (A) *Utx^fl/Δ^*;*Jmjd3^fl/Δ^*;Vasa*Cre* female mice (with oocytes carrying deletion *Utx* and *Jmjd3*) were crossed with *Utx^fl/y^*;*Jmjd3^fl/Δ^*;Vasa*Cre* male mice (with sperm carrying deletion of *Utx* and *Jmjd3*) and embryos from the cross were dissected at E10. *Utx^Δ/y^*;*Jmjd3^Δ/Δ^* (A′) and *Utx^Δ/Δ^*;*Jmjd3^Δ/Δ^* (A″) embryos from this cross phenocopy embryos derived from crosses with maternal contribution of UTX and JMJD3 (Figure 1D,E). (B) Genotyping of *Utx* and *Jmjd3* of embryos derived in the cross in Figure S2A to demonstrate complete recombination of *Utx* and *Jmjd3* floxed (fl) alleles to deletions (Δ).
(TIF)

Figure S3 Analysis of mid-gestation *Utx^Δ/y^*;*Jmjd3^Δ/Δ^* male embryos. (A,B) Quantitative RT-PCR of KDM6 members (A, *Utx* = light grey, *Jmjd3* = dark grey, *Uty* = black) and indicated *Hox* genes (B, *Hoxc4* = light grey, *Hoxa6* = dark grey, *Hoxa13* = black) in all indicated combinations of E10.5 male *Utx* and *Jmjd3* mutant embryos relative to *Utx^+/y^*;*Jmjd3^+/Δ^* controls (N≥4 samples per genotype). (C) Histones were extracted from *Utx^+/y^*;*Jmjd3^+/Δ^* or *Utx^Δ/y^*;*Jmjd3^Δ/Δ^* embryos and fluorescent western blots are illustrated for H3K27me3 relative to total H3 loading control. (D) Quantitation of the western blot in part C, H3k27me3 values were normalized to total H3 to calculate H3K27me3%. (E) H3K27me3 immunofluorescence of E10.5 *Utx^+/y^*;*Jmjd3^+/Δ^* or *Utx^Δ/y^*;*Jmjd3^Δ/Δ^* myocardium. (F) H3K27me3 immunofluorescence of E10.5 *Utx^+/y^*;*Jmjd3^+/Δ^* or *Utx^Δ/y^*;*Jmjd3^Δ/Δ^* ISL1 positive motor neurons in the proximal spinal chord. (G) Histones were extracted from *Utx^+/y^*;*Jmjd3^+/Δ^* or *Utx^Δ/y^*;*Jmjd3^Δ/Δ^* MEF lines and fluorescent western blots are illustrated for H3K27me3 relative to total H4 loading control. (H) Quantitation of western blots for H3K27me3%, from *Utx^+/y^*;*Jmjd3^+/Δ^* (grey) or *Utx^Δ/y^*;*Jmjd3^Δ/Δ^* (black) MEFs relative to total H4. Significant increases in protein levels are indicated (* p-value = 0.05).
(TIF)

Figure S4 Efficiency of tamoxifen *Cre^ER^* induction in *Utx* and *Jmjd3* floxed ES cell lines. (A) PCR genotyping of a *Utx^fl/fl^*;*Jmjd3^fl/fl^*;*Cre^ER^* ES cell line left untreated (−TX) or treated with tamoxifen for 2 days (+TX) for *Utx*, *Jmjd3*, Sex, and presence of Cre. Controls PCR reactions included are *Utx* or *Jmjd3* floxed alleles (*fl*), *Utx* or *Jmjd3* deleted alleles (*Δ*), Male DNA (Male), Female DNA (Female), Cre negative DNA (Neg), Cre positive DNA (Pos), water (H). (B) Western blot of a *Utx^fl/y^*;*Jmjd3^fl/fl^*;*Cre^ER^* ES cell line left untreated (−TX) or treated with tamoxifen for 2 days (+TX) and the *Utx^GT1/y^* ES line for UTX (αUTX) or a loading control (αGAPDH). The *Utx^GT1/y^* line should trap all *Utx* products. Therefore, the band present in all lanes at 140 KD is a non-specific band rather than an alternative product because it is not reduced in the *Utx^GT1/y^* sample. (C) RT-PCR for the 3 lines described in part B across the *Utx* region deleted by the floxed allele (Exon 3) and the JmjC domain. Note that all transcripts containing the JmjC domain are reduced in the *Utx^GT1/y^* RNA. (D) Western blot using a second independent UTX antibody.
(TIF)

Figure S5 Hanging drop EB analysis and constitutive deletion of *Utx* and *Jmjd3*. (A) As an alternative to formation of EBs in mass culture of ES cells, hanging drop EBs were generated from a defined cell number in a defined drop volume. (B) Fluorescent western blot analyzing loss of H3K27me3 with EB differentiation. H3K27me3 is blotted relative to total H4. (C) Quantification of the blot in Figure S5B. H3K27me3% is plotted relative to total H4 (D) Differentiation following constitutive long term deletion of *Utx* and *Jmjd3*. *Utx^fl/y^*;*Jmjd3^fl/fl^*;*Cre^ER^* ES cells were treated with TX for 3 days, then plated at low density to allow picking of single cell colonies. A clone of *Utx^Δ/y^*;*Jmjd3^Δ/Δ^* cells was propagated over 3 weeks and several passages, then differentiated into a typical day 4 EB structure relative to the parental *Utx^fl/y^*;*Jmjd3^fl/fl^*;*Cre^ER^* line. (E) Expression analysis of the ES cells (diff −) and day 8 EBs (diff +) described in Figure S5D for *Utx*, *Jmjd3*, *Gata6* (endoderm), and *Flk1* (Mesoderm). (F) *Utx^fl/fl^*;*Jmjd3^fl/fl^*;*Cre^ER^* ES cells were also treated with TX to generate single cell clones (as described in Figure S5D) and *Utx^Δ/Δ^*;*Jmjd3^Δ/Δ^* ES cells (Diff −) were differentiated with RA for 2 days (diff +) and RT-PCR compared expression of *Utx*, *Jmjd3*, *Hoxa1*, and *Hoxb1* relative to the parental *Utx^fl/fl^*;*Jmjd3^fl/fl^*;*Cre^ER^* line.
(TIF)

Figure S6 UCSC genome browser view of *Hoxb*, *Hoxc*, and *Hoxd* clusters. (A–C) UCSC genome browser view of *Hoxb* (A), *Hoxc* (B), and *Hoxd* (C), clusters. Illustrated are Input (black), WT ES H3K27me3 ChIP (dark green), KO ES H3K27me3 ChIP (dark red), WT RA H3K27me3 ChIP (light green), KO RA H3K27me3 ChIP (light red), WT RA H3K4me3 ChIP (blue), KO RA H3K4me3 ChIP (orange), and MACS defined enrichment peaks are illustrated as black bars underneath each track.
(TIF)

Figure S7 Analysis of MLL and PCR2 complexes in *Utx* and *Jmjd3* mutant cells. (A) Western blot analyzing levels of MLL complex members ASH2L and RBBP5 in *Utx^fl/fl^*;*Jmjd3^fl/fl^*;*Cre^ER^* ES cells −TX or +TX. (B) Western blot levels of PRC2 component EZH2 and RBBP5 in WT ES cells, 2 day RA differentiated ES cells, or 2 day differentiated EBs. (C) EZH2 ChIP-qPCR of *Utx^fl/fl^*;*Jmjd3^fl/fl^*;*Cre^ER^* ES cells (dark green, Diff −) or after 2 days of RA treatment (light green or red, Diff +) left untreated (green) or pre-treated with tamoxifen (red). An IgG control ChIP is illustrated as white bars. Quantitative PCR of an EZH2 negative locus (*Npm1*, *Neg*) was utilized for comparison to *RARE*, *Hoxa1*, *Hoxa2*, *Hoxb1*, *Hoxb2*, and *Hoxd9*.
(TIF)

Figure S8 Analysis of *Utx* and *Jmjd3* mutant MEFs. (A) RT-PCR of *Hoxa3*, *Hoxc4*, *Hoxa13*, or *Hoxd13* in ES cells, retinoic acid treated ES cells, or MEFs. (B) Western blot of EZH2 relative to RBBP5 loading control from *Utx^+/y^*;*Jmjd3^+/Δ^* or *Utx^Δ/y^*;*Jmjd3^Δ/Δ^* MEFs. (C) Quantitation of western blots for EZH2 or H3K4me3 from *Utx^+/y^*;*Jmjd3^+/Δ^* (grey) or *Utx^Δ/y^*;*Jmjd3^Δ/Δ^* (black) MEFs. Significant increases in protein levels are indicated (*p-value = 0.04).
(TIF)

Table S1 Promoters experiencing H3K27me3 loss in ES to RA differentiation. EdgeR statistical comparison for significant (FDR<0.05) loss of H3K27me3 normalized sequence reads in WT RA relative to WT ES (sheet 1) and KO RA relative to KO ES (Sheet 2) across the TSS +/− 1 KB. Some genes have multiple alternative promoters, so genes that are duplicated within the list are highlighted in red. Also denoted is whether the promoter has a corresponding RA H3K4me3 MACS peak (listed as 1).
(XLSX)

Table S2 Promoters experiencing H3K4me3 reduction in KO RA differentiation. EdgeR statistical comparison for significant (FDR<0.05) loss of H3K4me3 normalized sequence reads in KO RA relative to WT RA across the TSS +/−1 KB. Some genes have multiple alternative promoters, so genes that are duplicated within the list are highlighted in red. Also denoted is whether the promoter has a corresponding WT or KO ES H3K27me3 MACS peak (listed as 1).
(XLSX)

Table S3 KO RA H3K27me3 MACS peaks that have enrichment in H3K27me3 sequence reads over WT RA. EdgeR statistical comparison for significant (FDR<0.05) increase in H3K27me3 normalized sequence reads in KO RA MACS peaks relative to WT RA across the KO RA peak center +/−0.5 KB. MACS peaks are listed with the nearest neighboring gene. Some genes are associated with multiple MACS peaks, so genes that are duplicated within the list are highlighted in red. Also denoted is whether the promoter of that gene has a corresponding reduction in KO RA H3K4me3 (listed as 1).
(XLSX)

Table S4 Primers used in the study.
(XLSX)
