# Supplementary figures and images for: KDM6 Demethylase Independent Loss of Histone H3 Lysine 27 Trimethylation during Early Embryonic Development
Source: PLoS Genet. 2014 Aug 7;10(8):e1004507. doi: 10.1371/journal.pgen.1004507 (PMC4125042; doi:10.1371/journal.pgen.1004507)

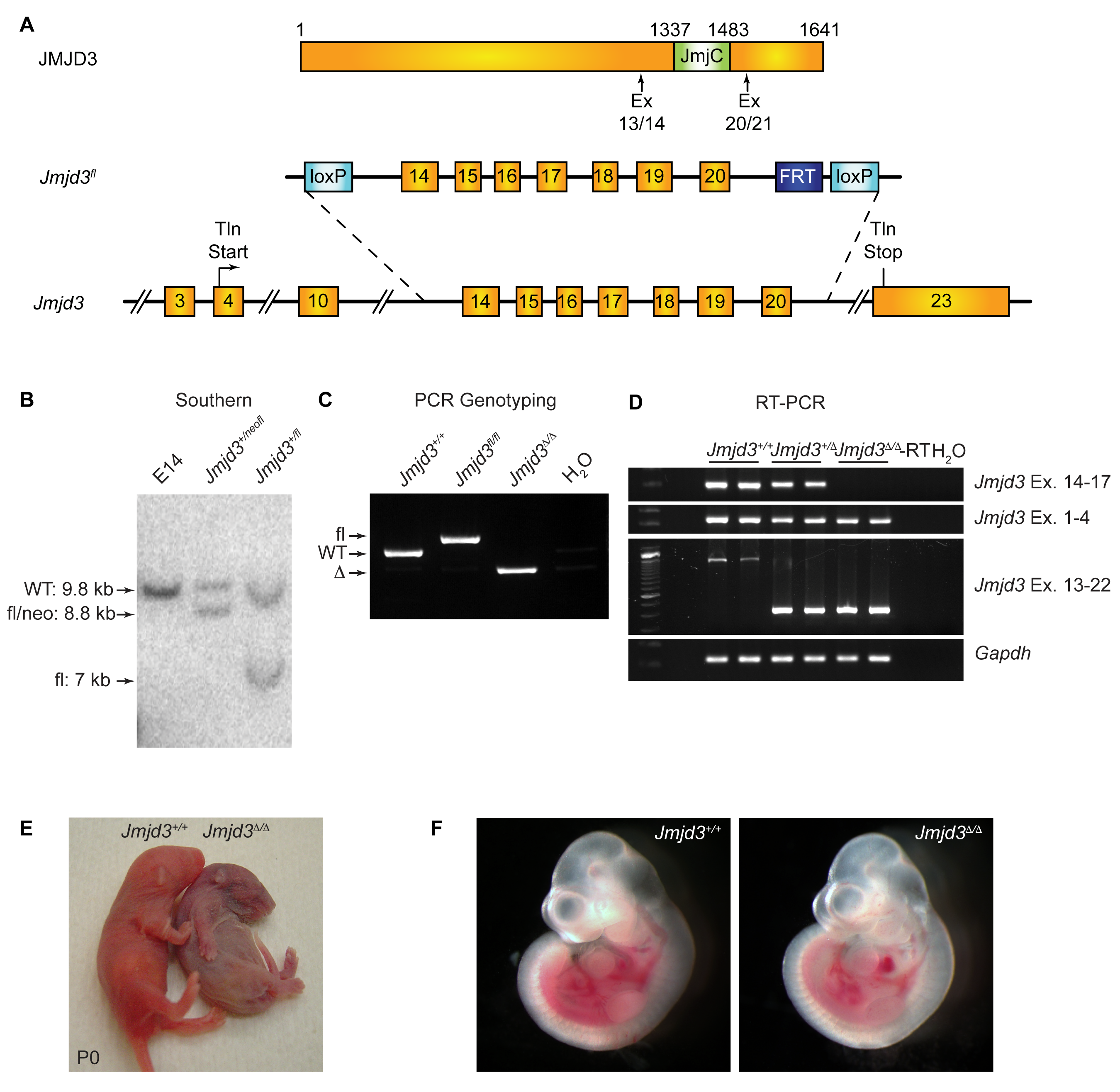

Supplement: Figure S1 — Targeted mutation of Jmjd3. (A) Jmjd3 was targeted to introduce a LoxP site in intron 13 and another in intron 20. The FRT site remains after the Neo cassette was removed by FLP recombinase. A schematic of the JMJD3 protein is illustrated above to denote the region that will be removed with Cre recombinase. Splicing from exon 13–21 will introduce a frameshift and stop codon. (B) Verification of the Jmjd3fl allele. Southern blotting of WT and targeted Jmjd3+/neofl ES cells with a 3′ probe and XmnI digest demonstrated the expected shift in banding due to a novel restriction site. The neo cassette was removed from targeted ES cells by transfection of a Flp recombinase to create Jmjd3+/fl. (C) Jmjd3fl /fl mice were crossed to a germline Cre recombinase to create Jmjd3Δ/Δ. A PCR genotyping scheme was designed to distinguish WT, Jmjd3fl, and Jmjd3Δ alleles in resulting mice. (D) The Jmjd3 deletion is verified by RT-PCR of E18.5 whole embryo RNA. Jmjd3Δ/Δ embryos lack the exons 14–17 product and amplification across exons 13–22 produces a smaller band that corresponds to the expected product lacking exons 14–20. Therefore, the mutant transcript is stable and is expected to produce JMJD3 lacking the JmjC histone demethylase domain. (E) Homozygous Jmjd3 mutant embryos are born, but fail to breathe as indicated by their blue color. (F) Jmjd3Δ/Δ embryos appear phenotypically normal at E10.5. (TIF) [file pgen.1004507.s001.tif]

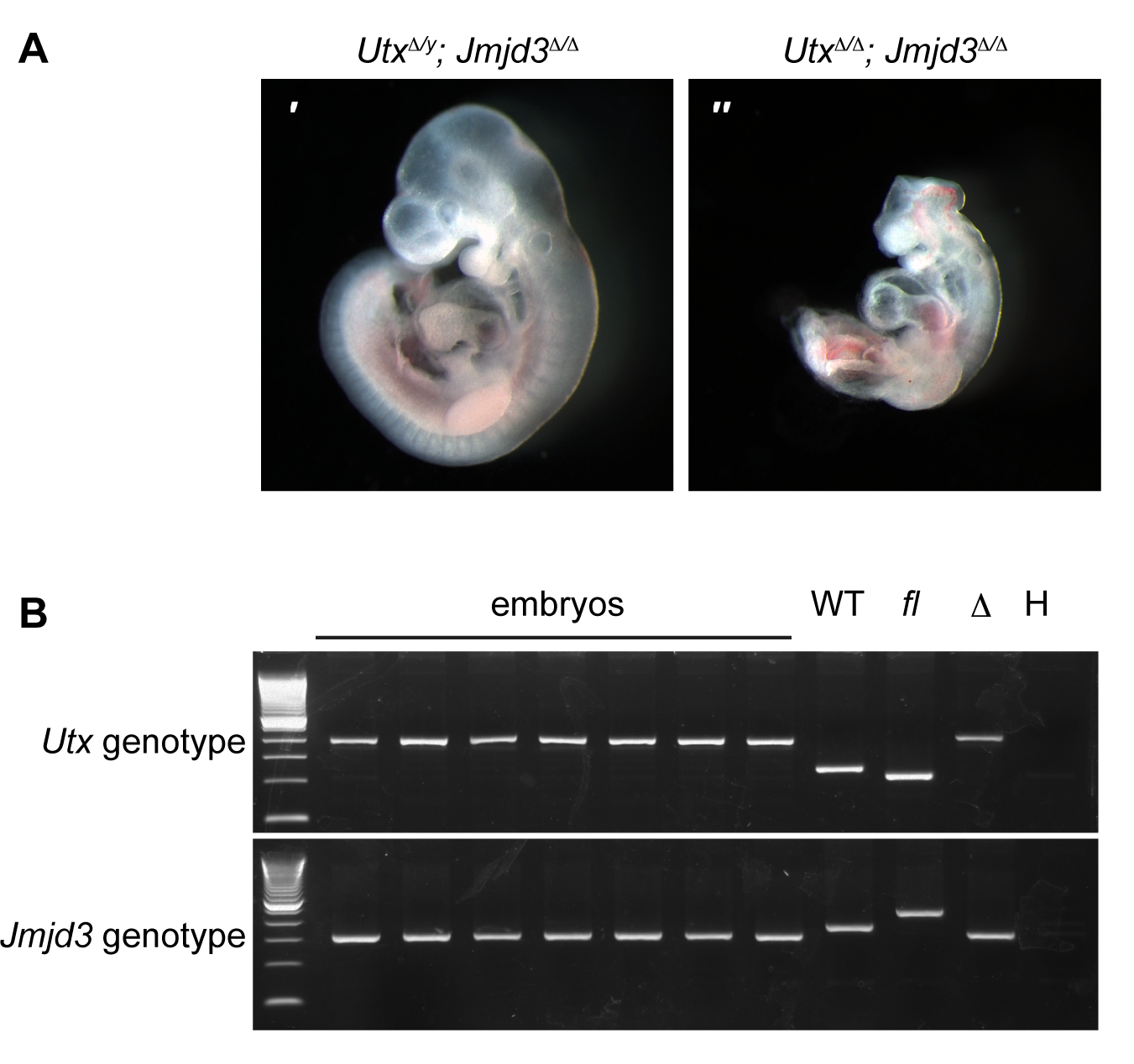

Supplement: Figure S2 — Maternal deletion of UTX and JMJD3 does not enhance embryonic mutant phenotypes. (A) Utxfl/Δ;Jmjd3fl/Δ;VasaCre female mice (with oocytes carrying deletion Utx and Jmjd3) were crossed with Utxfl/y;Jmjd3fl/Δ;VasaCre male mice (with sperm carrying deletion of Utx and Jmjd3) and embryos from the cross were dissected at E10. UtxΔ/y;Jmjd3Δ/Δ (A′) and UtxΔ/Δ;Jmjd3Δ/Δ (A″) embryos from this cross phenocopy embryos derived from crosses with maternal contribution of UTX and JMJD3 (Figure 1D,E). (B) Genotyping of Utx and Jmjd3 of embryos derived in the cross in Figure S2A to demonstrate complete recombination of Utx and Jmjd3 floxed (fl) alleles to deletions (Δ). (TIF) [file pgen.1004507.s002.tif]

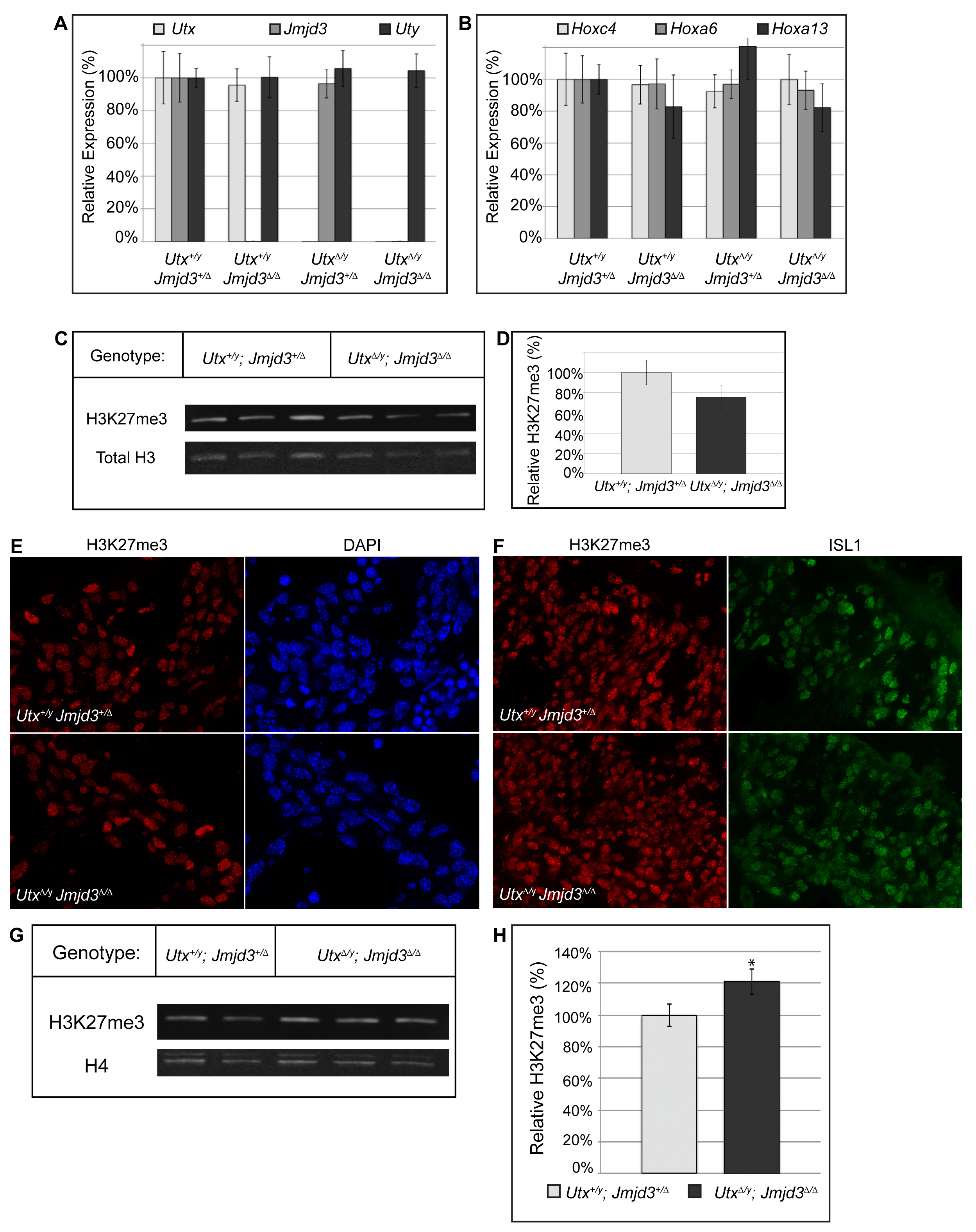

Supplement: Figure S3 — Analysis of mid-gestation UtxΔ/y;Jmjd3Δ/Δ male embryos. (A,B) Quantitative RT-PCR of KDM6 members (A, Utx = light grey, Jmjd3 = dark grey, Uty = black) and indicated Hox genes (B, Hoxc4 = light grey, Hoxa6 = dark grey, Hoxa13 = black) in all indicated combinations of E10.5 male Utx and Jmjd3 mutant embryos relative to Utx+/y;Jmjd3+/Δ controls (N≥4 samples per genotype). (C) Histones were extracted from Utx+/y;Jmjd3+/Δ or UtxΔ/y;Jmjd3Δ/Δ embryos and fluorescent western blots are illustrated for H3K27me3 relative to total H3 loading control. (D) Quantitation of the western blot in part C, H3k27me3 values were normalized to total H3 to calculate H3K27me3%. (E) H3K27me3 immunofluorescence of E10.5 Utx+/y;Jmjd3+/Δ or UtxΔ/y;Jmjd3Δ/Δ myocardium. (F) H3K27me3 immunofluorescence of E10.5 Utx+/y;Jmjd3+/Δ or UtxΔ/y;Jmjd3Δ/Δ ISL1 positive motor neurons in the proximal spinal chord. (G) Histones were extracted from Utx+/y;Jmjd3+/Δ or UtxΔ/y;Jmjd3Δ/Δ MEF lines and fluorescent western blots are illustrated for H3K27me3 relative to total H4 loading control. (H) Quantitation of western blots for H3K27me3%, from Utx+/y;Jmjd3+/Δ (grey) or UtxΔ/y;Jmjd3Δ/Δ (black) MEFs relative to total H4. Significant increases in protein levels are indicated (* p-value = 0.05). (TIF) [file pgen.1004507.s003.tif]

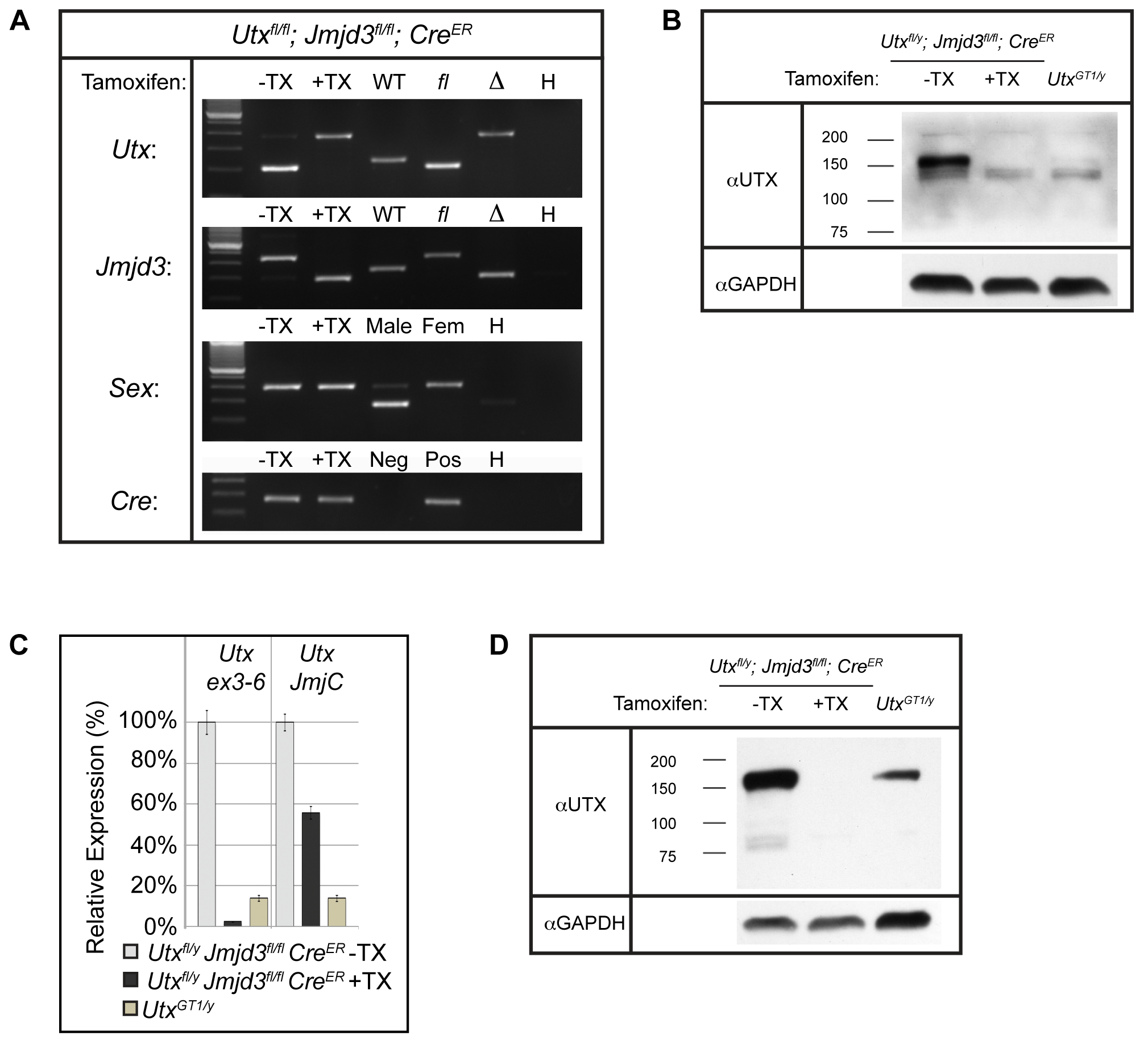

Supplement: Figure S4 — Efficiency of tamoxifen CreER induction in Utx and Jmjd3 floxed ES cell lines. (A) PCR genotyping of a Utxfl/fl;Jmjd3fl/fl;CreER ES cell line left untreated (−TX) or treated with tamoxifen for 2 days (+TX) for Utx, Jmjd3, Sex, and presence of Cre. Controls PCR reactions included are Utx or Jmjd3 floxed alleles (fl), Utx or Jmjd3 deleted alleles (Δ), Male DNA (Male), Female DNA (Female), Cre negative DNA (Neg), Cre positive DNA (Pos), water (H). (B) Western blot of a Utxfl/y;Jmjd3fl/fl;CreER ES cell line left untreated (−TX) or treated with tamoxifen for 2 days (+TX) and the UtxGT1/y ES line for UTX (αUTX) or a loading control (αGAPDH). The UtxGT1/y line should trap all Utx products. Therefore, the band present in all lanes at 140 KD is a non-specific band rather than an alternative product because it is not reduced in the UtxGT1/y sample. (C) RT-PCR for the 3 lines described in part B across the Utx region deleted by the floxed allele (Exon 3) and the JmjC domain. Note that all transcripts containing the JmjC domain are reduced in the UtxGT1/y RNA. (D) Western blot using a second independent UTX antibody. (TIF) [file pgen.1004507.s004.tif]

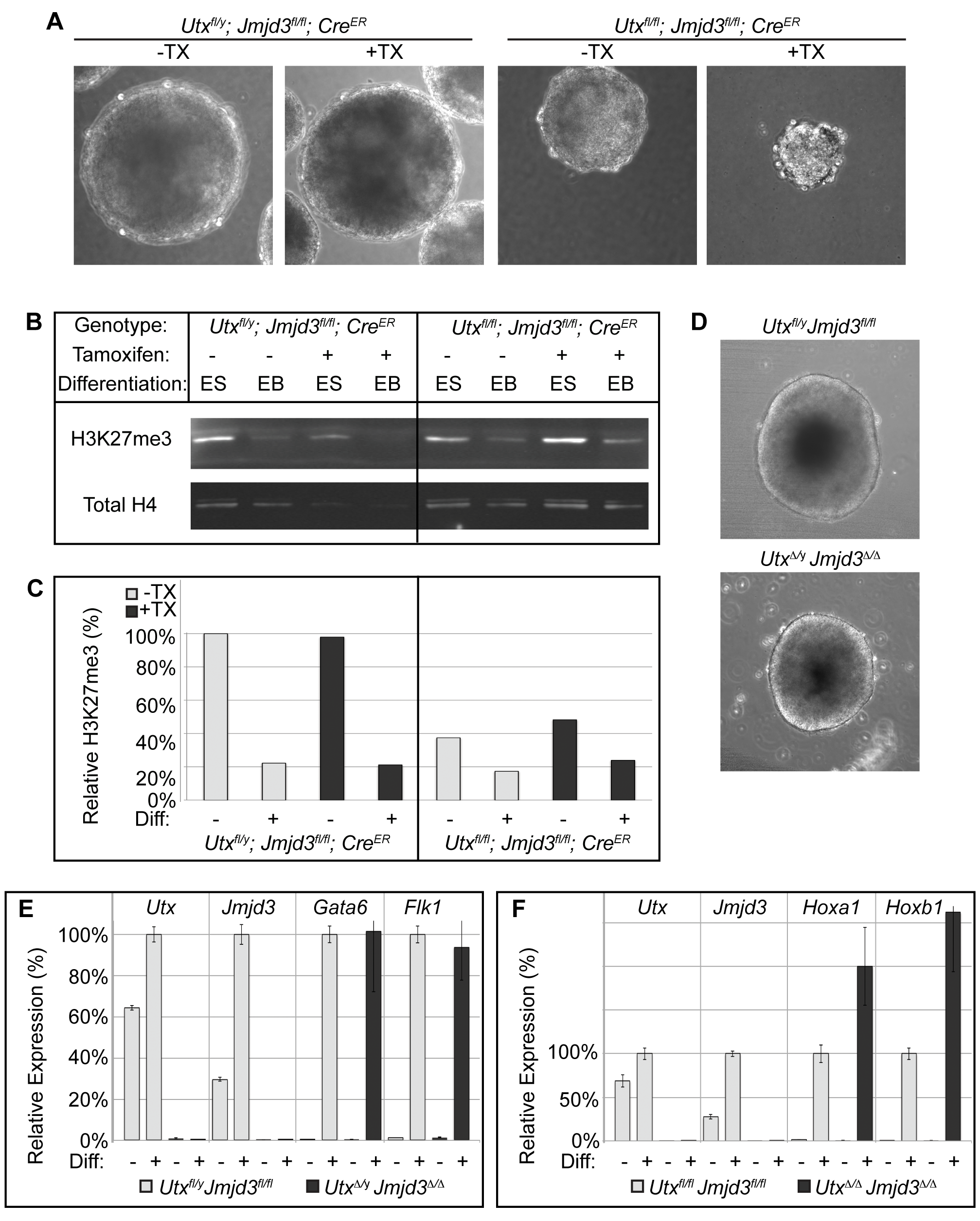

Supplement: Figure S5 — Hanging drop EB analysis and constitutive deletion of Utx and Jmjd3. (A) As an alternative to formation of EBs in mass culture of ES cells, hanging drop EBs were generated from a defined cell number in a defined drop volume. (B) Fluorescent western blot analyzing loss of H3K27me3 with EB differentiation. H3K27me3 is blotted relative to total H4. (C) Quantification of the blot in Figure S5B. H3K27me3% is plotted relative to total H4 (D) Differentiation following constitutive long term deletion of Utx and Jmjd3. Utxfl/y;Jmjd3fl/fl;CreER ES cells were treated with TX for 3 days, then plated at low density to allow picking of single cell colonies. A clone of UtxΔ/y;Jmjd3Δ/Δ cells was propagated over 3 weeks and several passages, then differentiated into a typical day 4 EB structure relative to the parental Utxfl/y;Jmjd3fl/fl;CreER line. (E) Expression analysis of the ES cells (diff −) and day 8 EBs (diff +) described in Figure S5D for Utx, Jmjd3, Gata6 (endoderm), and Flk1 (Mesoderm). (F) Utxfl/fl;Jmjd3fl/fl;CreER ES cells were also treated with TX to generate single cell clones (as described in Figure S5D) and UtxΔ/Δ;Jmjd3Δ/Δ ES cells (Diff −) were differentiated with RA for 2 days (diff +) and RT-PCR compared expression of Utx, Jmjd3, Hoxa1, and Hoxb1 relative to the parental Utxfl/fl;Jmjd3fl/fl;CreER line. (TIF) [file pgen.1004507.s005.tif]

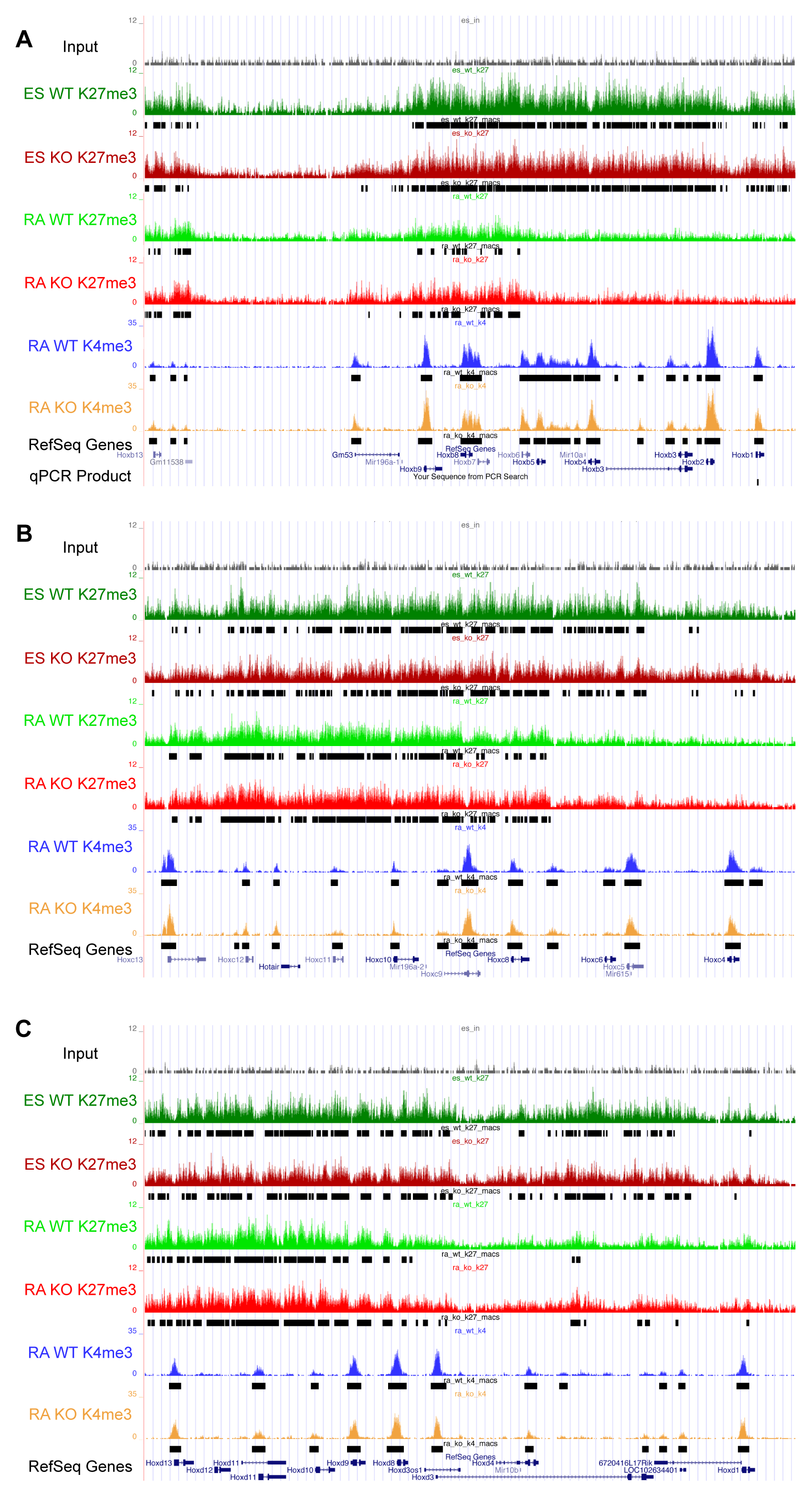

Supplement: Figure S6 — UCSC genome browser view of Hoxb, Hoxc, and Hoxd clusters. (A–C) UCSC genome browser view of Hoxb (A), Hoxc (B), and Hoxd (C), clusters. Illustrated are Input (black), WT ES H3K27me3 ChIP (dark green), KO ES H3K27me3 ChIP (dark red), WT RA H3K27me3 ChIP (light green), KO RA H3K27me3 ChIP (light red), WT RA H3K4me3 ChIP (blue), KO RA H3K4me3 ChIP (orange), and MACS defined enrichment peaks are illustrated as black bars underneath each track. (TIF) [file pgen.1004507.s006.tif]

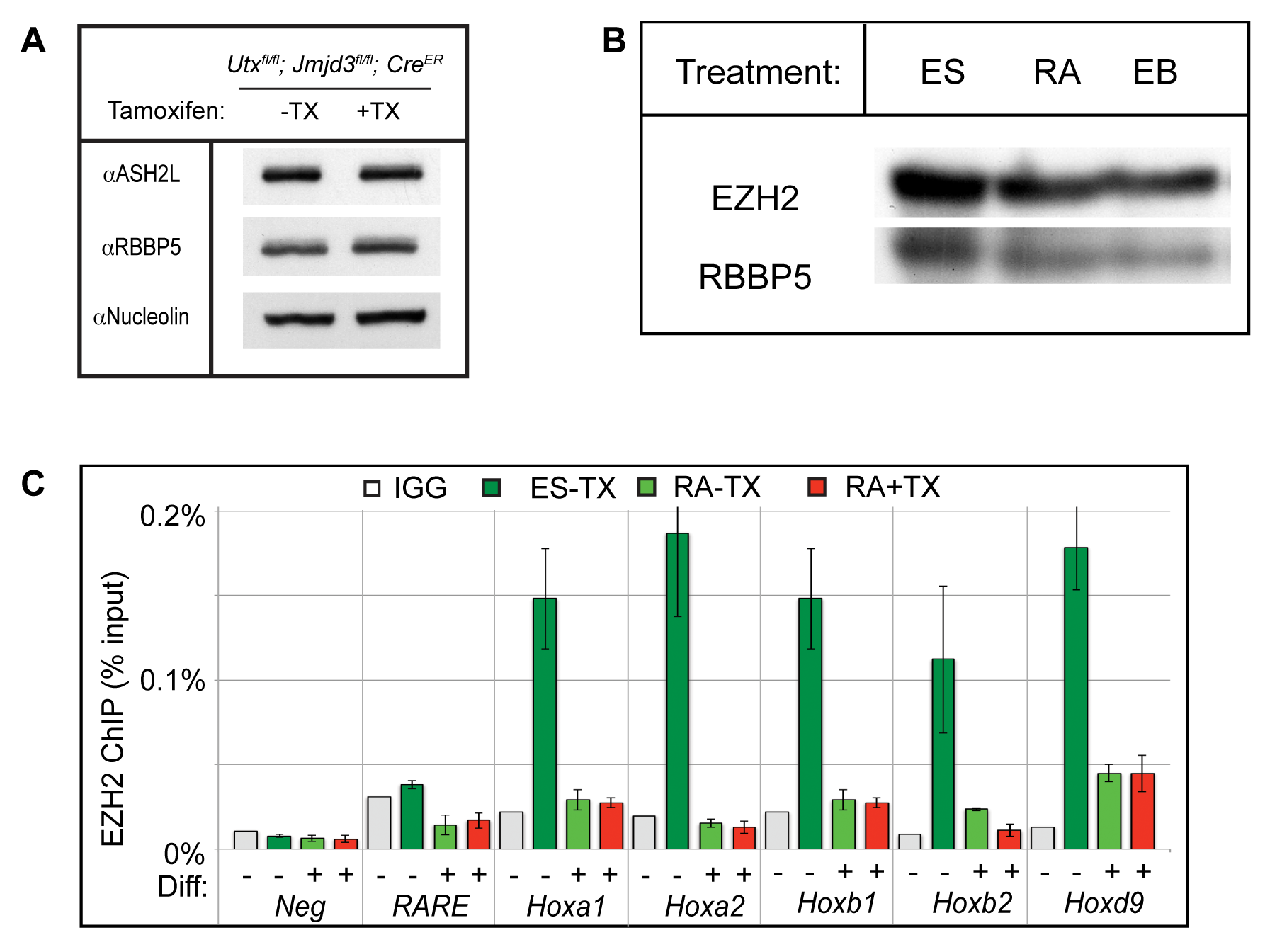

Supplement: Figure S7 — Analysis of MLL and PCR2 complexes in Utx and Jmjd3 mutant cells. (A) Western blot analyzing levels of MLL complex members ASH2L and RBBP5 in Utxfl/fl;Jmjd3fl/fl;CreER ES cells −TX or +TX. (B) Western blot levels of PRC2 component EZH2 and RBBP5 in WT ES cells, 2 day RA differentiated ES cells, or 2 day differentiated EBs. (C) EZH2 ChIP-qPCR of Utxfl/fl;Jmjd3fl/fl;CreER ES cells (dark green, Diff −) or after 2 days of RA treatment (light green or red, Diff +) left untreated (green) or pre-treated with tamoxifen (red). An IgG control ChIP is illustrated as white bars. Quantitative PCR of an EZH2 negative locus (Npm1, Neg) was utilized for comparison to RARE, Hoxa1, Hoxa2, Hoxb1, Hoxb2, and Hoxd9. (TIF) [file pgen.1004507.s007.tif]

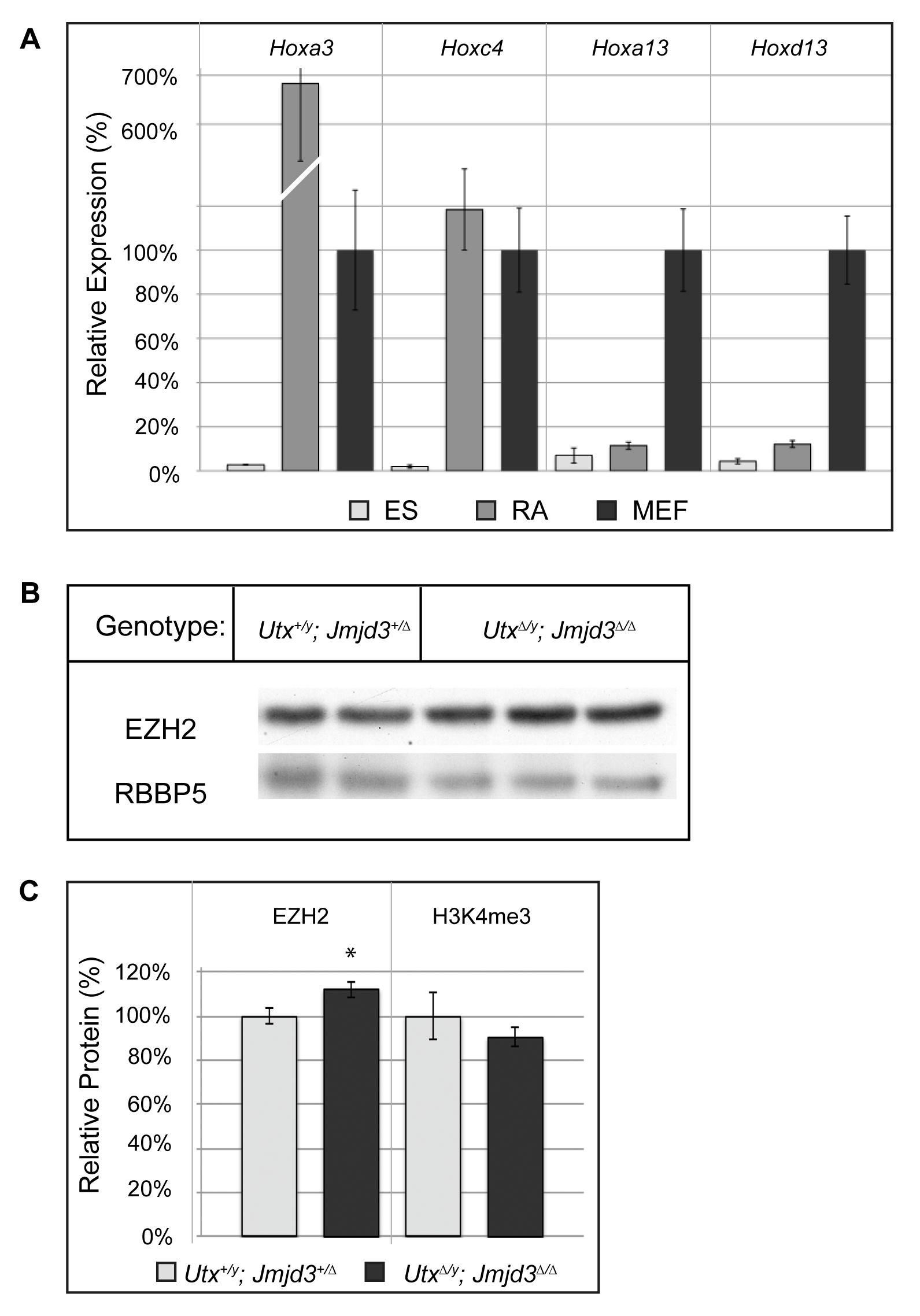

Supplement: Figure S8 — Analysis of Utx and Jmjd3 mutant MEFs. (A) RT-PCR of Hoxa3, Hoxc4, Hoxa13, or Hoxd13 in ES cells, retinoic acid treated ES cells, or MEFs. (B) Western blot of EZH2 relative to RBBP5 loading control from Utx+/y;Jmjd3+/Δ or UtxΔ/y;Jmjd3Δ/Δ MEFs. (C) Quantitation of western blots for EZH2 or H3K4me3 from Utx+/y;Jmjd3+/Δ (grey) or UtxΔ/y;Jmjd3Δ/Δ (black) MEFs. Significant increases in protein levels are indicated (*p-value = 0.04). (TIF) [file pgen.1004507.s008.tif]
